# LncRNA IFITM4P promotes immune escape by up-regulating PD-L1 via dual mechanism in oral carcinogenesis

**DOI:** 10.1016/j.ymthe.2022.01.003

**Published:** 2022-01-17

**Authors:** Linjun Shi, Yuquan Yang, Mengying Li, Chenxi Li, Zengtong Zhou, Guoyao Tang, Lan Wu, Yilin Yao, Xuemin Shen, Zhaoyuan Hou, Hao Jia

**Affiliations:** 1 Department of Oral Mucosal Diseases, Shanghai Ninth People's Hospital, Shanghai Jiao Tong University School of Medicine, College of Stomatology, Shanghai Jiao Tong University, Shanghai 200011, China; 2 National Center for Stomatology, National Clinical Research Center for Oral Diseases, Shanghai Key Laboratory of Stomatology, Shanghai, China; 3 Faculty of Basic Medicine, Shanghai Jiao Tong University School of Medicine, 280 South Chongqing Road, Shanghai 200025, China; 4 Shanghai Key Laboratory for Tumor Microenvironment and Inflammation, Department of Biochemistry & Molecular Cellular Biology, Shanghai Jiao Tong University School of Medicine, Shanghai 200025, China; 5 Key Laboratory of Cell Differentiation and Apoptosis of Chinese Ministry of Education, Faculty of Basic Medicine, Shanghai Jiao Tong University School of Medicine, Shanghai 200025, China

**Keywords:** oral squamous cell carcinoma, oral leukoplakia, immune escape, lncRNA, IFITM4P, PD-L1/PD-1, TAK1, NF-κB, SASH1, PTEN

## Abstract

Oral squamous cell carcinoma (OSCC), which is typically preceded by oral leukoplakia (OL), is a common malignancy with poor prognosis. However, the signaling molecules governing this progression remain to be defined. Based on microarray analysis of genes expressed in OL and OSCC samples, we discovered that the long non-coding RNA *IFITM4P* was highly expressed in OSCC, and ectopic expression or knockdown of IFITM4P resulted in increased or decreased cell proliferation *in vitro* and in xenografted tumors, respectively. Mechanistically, in the cytoplasm IFITM4P acted as a scaffold to facilitate recruiting SASH1 to bind and phosphorylate TAK1 (Thr187), and in turn to increase the phosphorylation of nuclear factor κB (Ser536) and concomitant induction of *PD-L1* expression, resulting in activation of an immunosuppressive program that allows OL cells to escape anti-cancer immunity in cytoplasm. In nucleus, IFITM4P reduced *Pten* transcription by enhancing the binding of KDM5A to the *Pten* promoter, thereby upregulating PD-L1 in OL cells. Moreover, mice bearing tumors with high IFITM4P expression had notable therapeutic sensitivity to PD-1 monoclonal antibody (mAb) treatment. Collectively, these data demonstrate that IFITM4P may serve as a new therapeutic target in blockage of oral carcinogenesis, and PD-1 mAb can be an effective reagent to treat OSCC.

## Graphical abstract

**Figure:**
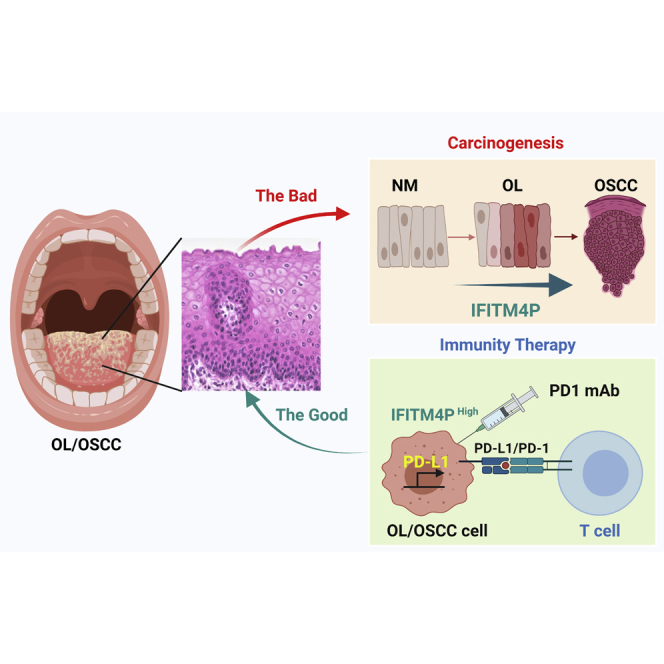

## Teaser

A novel lncRNA, IFITM4P, may serve as a new therapeutic target in blockage of oral carcinogenesis, and PD-1 mAb can be an effective reagent to treat OSCC with high expression of IFITM4P.

## Introduction

Oral squamous cell carcinoma (OSCC) is a common malignancy with poor prognosis, with 250,000 cases recorded annually.^1^, ^2^, ^3^ Oral leukoplakia (OL), as a type of oral potentially malignant disorder (OPMD), is an important clinical model for understanding oral carcinogenesis^4^ and is characterized by oral precancerous lesions.^5^ The optimal clinical management of OL remains unsatisfactory,^5^ with no effective treatment to prevent oral carcinogenesis.^6^ The development of OSCC from OL is a typical multistep carcinogenesis process. However, effective markers to predict oral carcinogenesis and by which mechanisms remain unclear, and their identification can help determine effective targets for blocking oral carcinogenesis and help improve the overall prognosis.^4^^,^^7^

Long non-coding RNAs (lncRNAs) are defined as RNA transcripts >200 nt in length that lack apparent protein-coding potential.^8^^,^^9^ However, many of them play important roles in various cellular processes in both normal and cancerous cells by controlling gene expression through versatile mechanisms. These include interaction with transcriptional factors and chromatin-modifying enzymes to regulate target genes directly or with proteins to regulate mRNA splicing, transportation, degradation, and translation. Previous bioinformatics analyses have shown the differential expression of certain lncRNAs during the occurrence and malignant transformation of OPMDs via serial analysis of gene expression;^10^, ^11^, ^12^ however, no key lncRNAs and their related mechanisms were validated by further experiments. Therefore, whether lncRNAs and related signal pathways play critical roles in oral carcinogenesis remains unclarified.

Immunosurveillance has been broadened to the field of cancer immunoediting, which is a process consisting of three stages: elimination, balance, and escape.^13^^,^^14^ Programmed cell death 1 ligand 1 (PD-L1) expressed on tumor cells inhibits the functional activity of cytotoxic lymphocytes and their ability to attack tumor cells, which is one of the mechanisms of immune evasion.^15^ Antibodies such as anti-PD-L1 have demonstrated broad applicability across cancer types and long-lasting clinical response when the treatment is effective.^16^ Recently, a newly published systematic review^17^ showed the expression of PD-L1 in the majority of OPMDs and OSCC samples, with expression levels correlating with increased progression and decreased survival rates. 4NQO-induced oral mucosal precancerous lesions in C57BL/6J mice serve as a common model for studying oral carcinogenesis.^18^ PD-1 monoclonal antibody (mAb) treatment can effectively inhibit the occurrence and development of oral precancerous lesions in this animal model.^19^, ^20^, ^21^ However, Wen et al. found that the PD-1 mAb was less than 70% effective in treating oral precancerous lesions.^22^ Levingston and Young found that the PD-1 mAb was temporarily effective in low-grade precancerous lesions and was significantly invalid in high-grade precancerous lesions to carcinoma *in situ*.^23^ These studies basically confirmed that there was immune escape at the stage of OL or precancerous lesions. Hence, elucidating the molecular mechanism of oral leukoplakia PD-L1 gene expression regulation can deepen the understanding of the mechanism of immune escape in the precancerous stage and is of great significance for clinical immune prevention and precision treatment.

In this paper, we report the discovery that the lncRNA IFITM4P is progressively induced from OL to OSCC cells through the lipopolysaccharide/Toll-like receptor 4 (LPS/TLR4) pathway and that high expression of IFITM4P results in increased OSCC cell proliferation and induction of *PD-L1* expression. Moreover, mice bearing tumors with high IFITM4P expression exhibit notably therapeutic sensitivity to PD-1 mAb treatment. Collectively, these data demonstrate that IFITM4P may serve as a new therapeutic target in blockage of oral carcinogenesis and that PD-1 mAb can be an effective reagent to treat OSCC with high expression of IFITM4P.

## Results

### Expression of lncRNA IFITM4P is increased with the progression of normal mucosa to OL and OSCC

**Figure 1:**
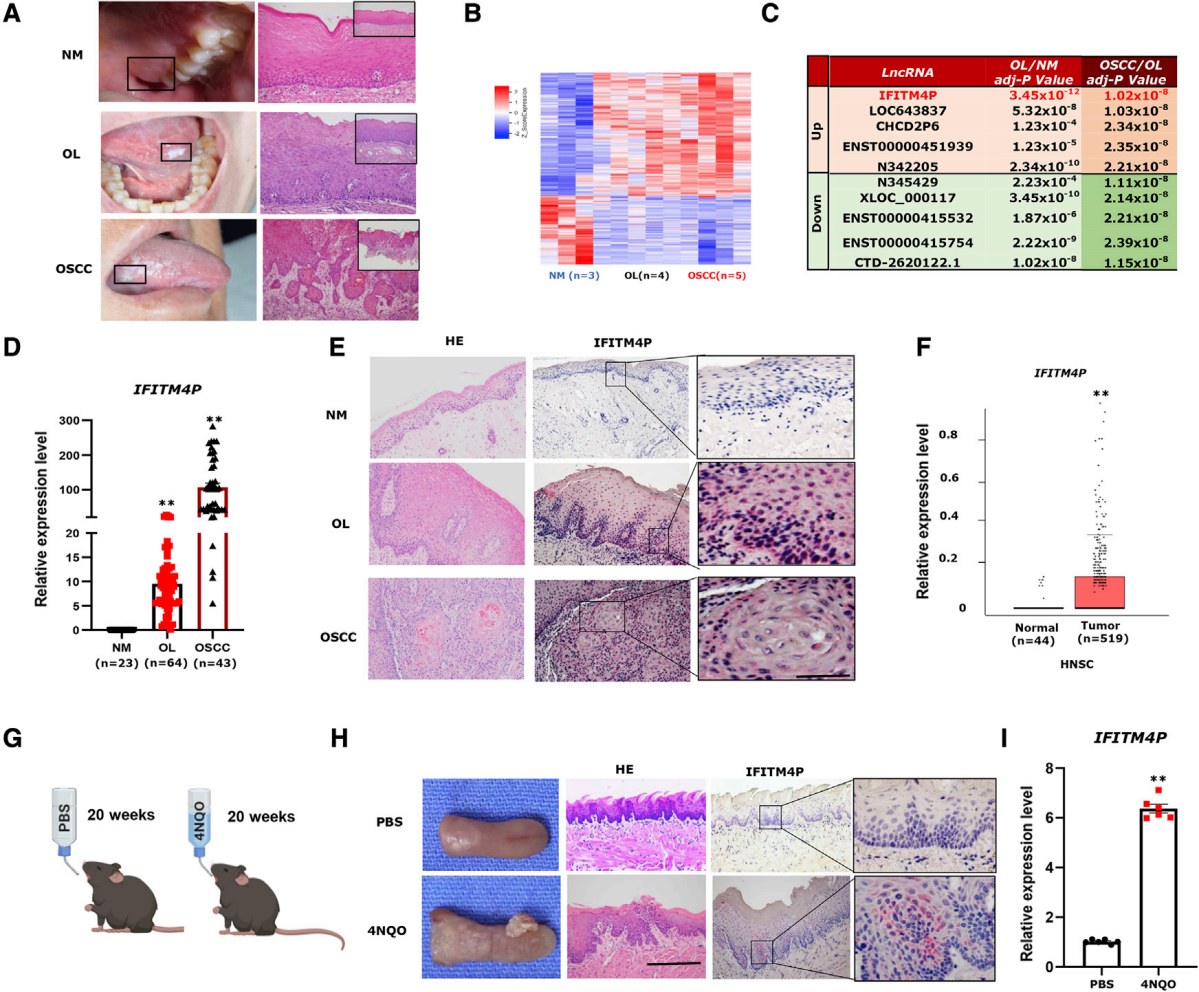
IFITM4P was upregulated in OL and OSCC compared with NM (A) Typical macroscopic and microscopic findings (H&E staining) in NM, OL, and OSCC (100×). (B) Hierarchical clustering microarray analysis was performed on NM (n = 3), OL (n = 4), and OSCC (n = 5) samples from Chinese patients based on differentially expressed RNA transcripts (p < 0.05, fold change >2) from the microarray data. Each column represents a sample and each row represents a transcript. The expression level of each gene in a single sample is depicted according to the color scale. (C) Identification of regulated lncRNAs in OL/NM and OSCC/OL and list of ten differentially expressed lncRNAs with fold change >2 and p < 0.001. (D) qRT-PCR analysis showed *IFITM4P* expression was highest in OSCC samples compared with OL and NM samples, with the lowest in NM samples, ∗∗p < 0.001. (E) In the stepwise samples from the same patient, IFITM4P staining became stronger during development of OSCC from OL (200×). IFITM4P staining appears negative in adjacent NM. (F) TCGA data show that higher expression of IFITM4P was seen in HNSC tissues (n = 519) than in normal tissues (n = 44), ∗∗p < 0.001. (G) Schematic timeline of the tongue leukoplakia/SCC model. (H) Typical tongue leukoplakia and SCC were found in the 4NQO group. The histopathological diagnosis also confirmed leukoplakia on the dorsal tongue and local early invasive tongue SCC in the 4NQO group. FISH showed strong IFITM4P staining in the 4NQO group, while no staining was found in the PBS group (n = 6). (I) qRT-PCR results showed that *IFITM4P* was increased in the 4NQO group compared with the PBS group. (n = 6), ∗∗p < 0.001.

To identify the differentially expressed lncRNAs in oral normal mucosa (NM), OL, and OSCC, a microarray analysis was performed on NM (n = 3), OL (n = 4), and OSCC (n = 5) samples from Chinese patients (Figure 1A and Table S1). The analysis revealed 3,109 interactions between lncRNAs and mRNA transcripts (p < 0.05, fold change >2) of the NM, OL, and OSCC groups. We focused on ten differentially expressed lncRNAs (five upregulated and five downregulated) with fold change >2 and ∗∗∗p < 0.0001 (Figure 1C). Notably, IFITM4P showed the highest expression in the OSCC/OL and OL/NM groups. To validate these observations, we performed qRT-PCR to examine the expression of IFITM4P in human NM (n = 23), OL (n = 64), and OSCC (n = 43) specimens. Consistently, the expression of IFITM4P was highest in OSCC samples compared with OL and NM samples, the lowest being in NM samples (Figure 1D). These results were validated using RNA fluorescence *in situ* hybridization (FISH) staining in OL, OSCC, and adjacent NM samples, respectively, from the same patients. In stepwise samples of the same patient, no *IFITM4P* was detected in the NM. However, as OL progressed to early invasive OSCC, *IFITM4P* staining became stronger (Figure 1E). Furthermore, data from The Cancer Genome Atlas (TCGA) indicated greater expression of IFITM4P in head and neck squamous carcinoma (HNSC) tissues (n = 519) than in normal tissues (n = 44) (∗∗p < 0.001) (Figure 1F). The OL/OSCC model induced by 4-nitroquinoline monoxide (4NQO) allows us to study the occurrence of oral epithelial cancer *in vivo*.^24^^,^^25^ The immunocompetent mice (C57BL/6) induced with 4NQO developed OL at 14–16 weeks and OSCC at 22–24 weeks.^26^ To evaluate the effect of IFITM4P on the development of OSCC from OL in mice induced with 4NQO, we used a modified C57/B6J mouse tongue leukoplakia/squamous cell carcinoma (SCC) model (Figure 1G). Macroscopically, no lesions were detected on the tongue from the mice in the PBS group (Figure 1H). Typical tongue leukoplakia and tumor were found in the 4NQO group (Figure 1H). To confirm the macroscopic findings, we performed H&E staining, qRT-PCR, and IFITM4P-FISH staining of the tongue lesions from two groups. The histopathological diagnosis also confirmed leukoplakia on the dorsal tongue and local early invasive tongue SCC in the 4NQO group (Figure 1H); FISH staining showed that IFITM4P in the 4NQO group was stronger, while mice in the PBS group were not stained (Figure 1H). Meanwhile, the qRT-PCR results showed that IFITM4P was increased in the 4NQO group compared with the PBS group (Figure 1I). Taken together, these results indicate that IFITM4P could be a biomarker during oral carcinogenesis.

### IFITM4P promotes the proliferation of OL and OSCC cells

**Figure 2:**
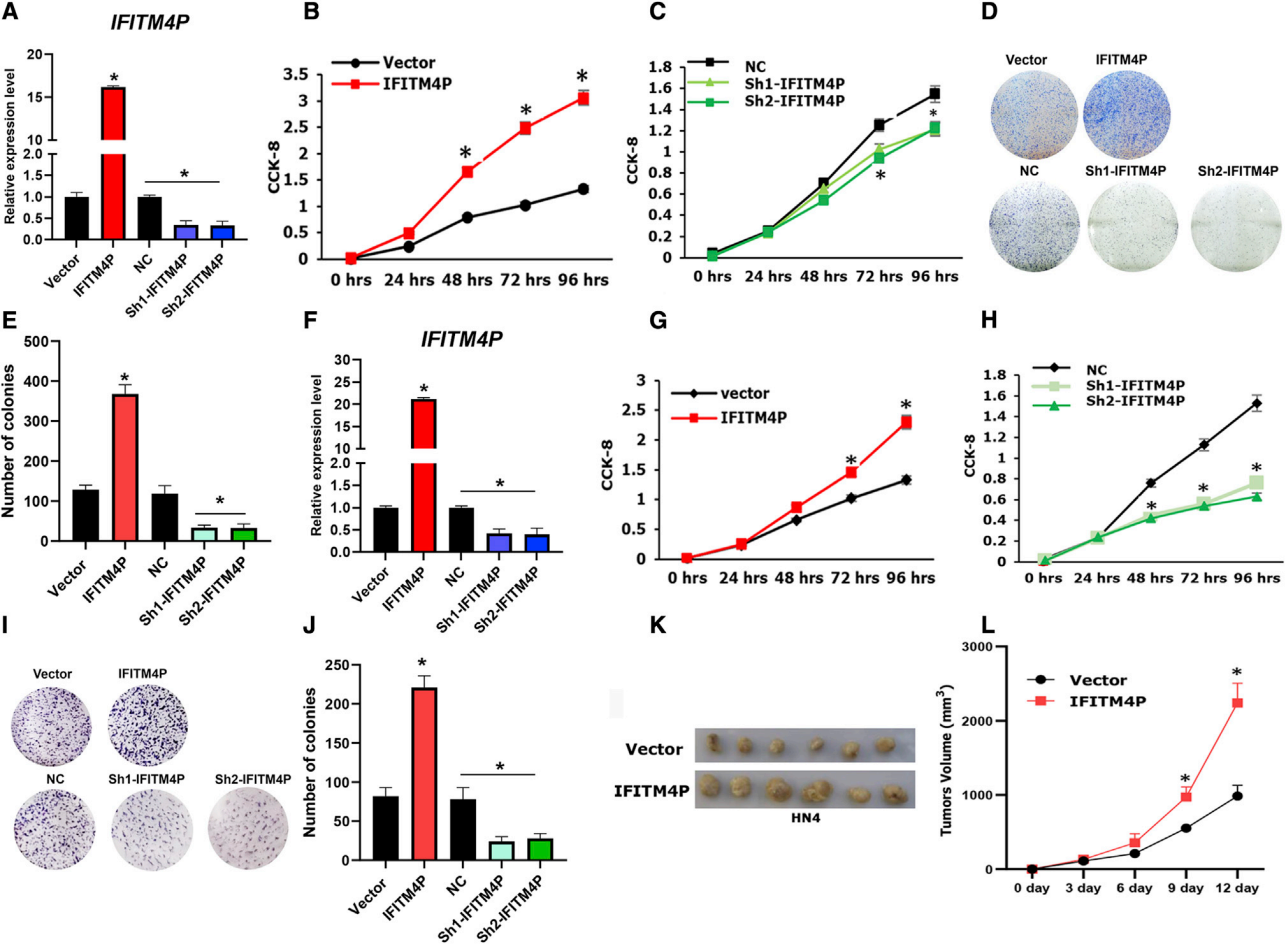
IFITM4P promotes OL and OSCC proliferation and colony formation (A) Stable overexpression of IFITM4P and knocked down in Leuk-1 cells using specific shRNAs via viral transductions; stable cells were established following puromycin selection. (B) CCK-8 assay showed that overexpression of IFITM4P significantly promoted cell proliferation Leuk-1. (C) CCK-8 assay showed that knockdown of IFITM4P significantly inhibited cell proliferation in Leuk-1. (D and E) Representative dishes (D) and quantification (E) show that overexpression of IFITM4P significantly increased cell colony formation and that knockdown inhibited cell colony formation in Leuk-1. (F) Stable overexpression of IFITM4P and knocked down in HN4 cells using specific shRNAs via viral transductions; stable cells were established following puromycin selection. (G) CCK-8 assay showed that overexpression of IFITM4P significantly promoted cell proliferation HN4. (H) CCK-8 assay showed that knockdown of IFITM4P significantly inhibited cell proliferation in HN4. (I and J) Representative dishes (I) and quantification (J) show that overexpression of IFITM4P significantly increased cell colony formation and knockdown inhibited cell colony formation in HN4. (K) IFITM4P promoted HN4 cell growth *in vivo* (n = 6). (L) Overexpression of IFITM4P in HN4 significantly increased tumor volume. Data from (A), (B), (C), (F), (G), and (H) are shown as mean ± SD from three independent experiments; ∗p < 0.05. Data from (E), (J), and (L) are shown as mean ± SD from six independent experiments; ∗p < 0.05.

To determine the role of IFITM4P in oral carcinogenesis, we chose Leuk-1 (OL)^27^ and HN4 (OSCC)^28^ cells. We manipulated IFITM4P expression via the stable transduction of Leuk-1 cells with lentiviral vectors carrying cDNA encoding the full length of IFITM4P (Figure 2A) and analyzed cell growth and colony formation in these cells. Our results showed that exogenous IFITM4P resulted in increased cell growth and colony formation in Leuk-1 cells (Figures 2B, 2D, and 2E). To test the converse, we depleted IFITM4P in Leuk-1 cells using IFITM4P-specific short hairpin RNA (shRNA) (Figure 2A) and observed a significant decrease in cell growth and colony formation (Figures 2B, 2D, and 2E). Similar results were seen in HN4 cells with overexpression (Figures 2F, 2G, 2I, and 2J) and depletion (Figures 2F, 2H, 2I, and 2J) of IFITM4P. To examine the role of IFITM4P in OSCC *in vivo*, we transplanted HN4-Vector and HN4-IFITM4P cells into BALB/C nude mice. IFITM4P overexpression in cells increased OSCC growth *in vivo* compared with the vector group (Figures 2K and 2L). Collectively, these data demonstrated that IFITM4P acts as a novel oncogene during oral carcinogenesis.

### PD-L1 is a target of IFITM4P in OL and OSCC

**Figure 3:**
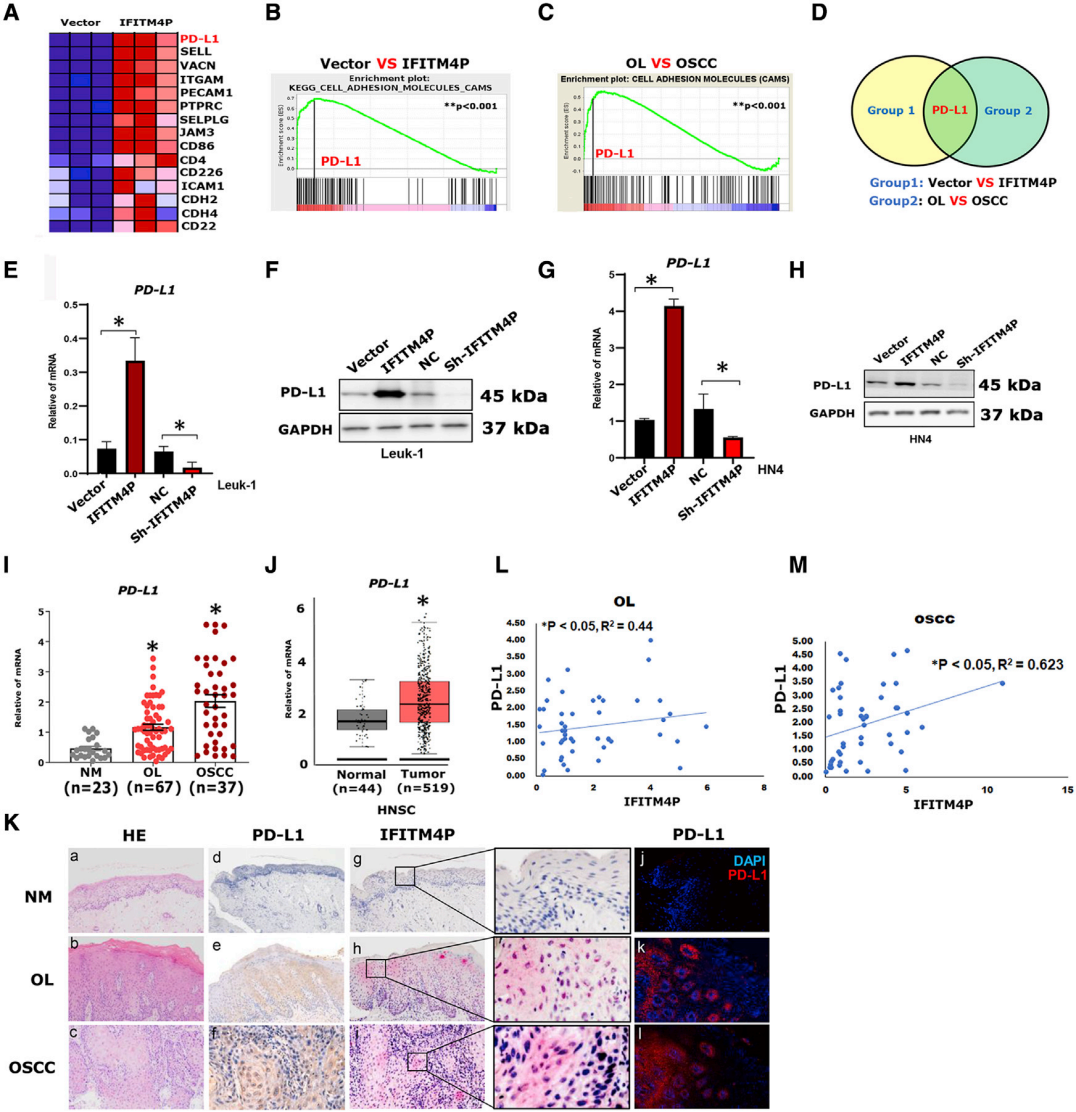
PD-L1 is a novel downstream target of IFITM4P in OL and OSCC (A) Heatmap of cell adhesion-associated genes (by GSEA analysis) in the leading edge showing the strongest upregulation in the IFITM4P group in Leuk-1 cells. (B) Comparison of the enrichment plots for vector versus IFITM4P-expressing Leuk-1 cells generated by GSEA analysis of ranked gene expression data (left, upregulated [red]; right, downregulated [blue]). The enrichment score is shown as a green line (enrichment score = 0.52; ∗∗p < 0.001). (C) Comparison of enrichment plots for OL versus OSCC generated by GSEA analysis of ranked gene expression data (left, upregulated [red]; right, downregulated [blue]). The enrichment score is shown as a green line (enrichment score = 0.49; ∗∗p < 0.001). (D) Venn diagram showing the overlap between vector versus IFITM4P-expressing Leuk-1 cells and OL versus OSCC. (E and F) Induction of PD-L1 by IFITM4P was confirmed by qRT-PCR (E) and WB (F) in Leuk-1 cells. (G and H) Induction of PD-L1 by IFITM4P was confirmed by qRT-PCR (G) and WB (H) in HN4 cells. (I) qRT-PCR analysis showed that *PD-L1* expression was highest in OSCC samples compared with OL and NM samples, with the lowest in NM samples. (J) TCGA data analysis showed higher PD-L1 expression in HNSC tissues (n = 519) than in normal tissues (n = 44), ∗p < 0.05. (K) In the samples from patients, no PD-L1 and IFITM4P staining was seen in NM. However, PD-L1 and IFITM4P staining became stronger as OL progressed to early invasive OSCC (a–l, 200×). (L and M) Positive correlation between the levels of IFITM4P and PL-D1 in OL (∗p < 0.05) (L) and OSCC (∗p < 0.05) (M) samples. Data from (E), (G), and (I) are shown as mean ± SD from three independent experiments. ∗p < 0.05.

To identify the downstream targets of IFITM4P that regulate the proliferation of Leuk-1 cells, we performed RNA sequencing (RNA-seq) to identify differentially expressed genes between overexpressed IFITM4P and vector (Figure 3A). Gene ontology analysis of the RNA-seq data indicated that IFITM4P affected many biological processes, including the immune response, innate immune response, and inflammatory response (Figure S1). Meanwhile, gene set enrichment analysis (GSEA) to reveal the gene signature regulated by IFITM4P showed enrichment of adhesion molecules (Figures 3B and 3C). We further compared the OL group with the OSCC group by GSEA (Figure 3D) and found PD-L1 to be significantly enriched in both groups. To validate the effect of IFITM4P on PD-L1 during oral carcinogenesis, we stably transduced Leuk-1 and HN4 cells with shRNA targeting IFITM4P or cDNA encoding the full-length IFITM4P. Induction of PD-L1 by IFITM4P was confirmed by qRT-PCR and western blot (WB) in Leuk-1 (Figures 3E and 3F) and HN4 cells (Figures 3G and 3H). To further evaluate the role of PD-L1 during oral carcinogenesis, we performed qRT-PCR assays on human NM (n = 23), OL (n = 67), and OSCC (n = 37) (Figure 3I) samples. qRT-PCR revealed a significantly higher expression of PD-L1 in OSCC samples compared with OL and NM samples (∗p < 0.05) (Figure 3I). Data from TCGA indicated greater expression of PD-L1 in HNSC tissues (n = 519) than in normal tissues (n = 44) (∗p < 0.05) (Figure 3J). Furthermore, we performed PD-L1 immunohistochemistry (IHC), PD-L1 immunofluorescence (IF), and *IFITM4P*-FISH staining on OL, OSCC, and NM samples, respectively. No PD-L1 and *IFITM4P* were detected in the NM. However, as OL progressed to early invasive OSCC, PD-L1 and *IFITM4P* staining became stronger (Figure 3K). Pearson's correlation analysis showed a positive correlation between IFITM4P and PD-L1 levels in both OL (Figure 3L, ∗p < 0.05, r^2^ = 0.443) and OSCC (Figure 3M, ∗p < 0.05, r^2^ = 0.623) samples. Collectively, these data demonstrate that PD-L1 is a new target of IFITM4P in OL and OSCC.

### IFITM4P/PD-L1 induced by LPS/TLR4 pathway promotes immune escape in mouse tongue carcinogenesis

**Figure 4:**
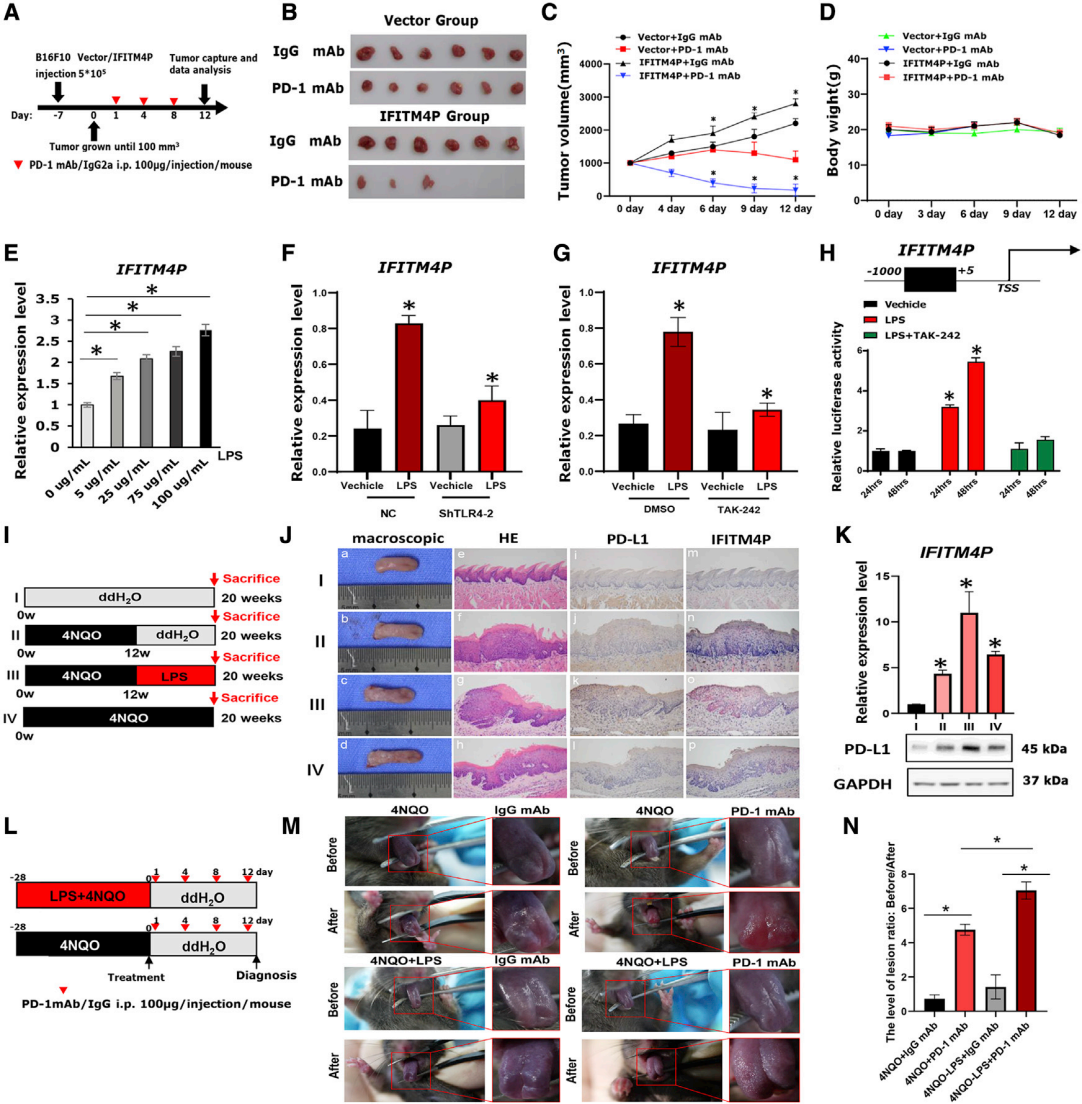
LPS/TLR4 activates IFITM4P/PD-L1 signaling pathway to promote immune escape (A) C57BL/6J mice were implanted with 5 × 10^5^ B16F10 cells expressing IFITM4P or vector and received PD-1 mAb or IgG isotype control (IgG2a) treatment. The timeline of tumor induction and treatment is shown. (B) Tumor volumes. (C) Introduction of IFITM4P in B16F10 cells significantly increased tumor volume in C57BL/6J mice. PD-1 mAb significantly reduced tumor volume in C57BL/6J mice bearing B16F10 cells expressing IFITM4P. (D) No significant difference in body weight of mice was found in each group. Mouse body weight was measured every 3 days. (E) qRT-PCR showed a dose-dependent increase in LPS-induced *IFITM4P* transcription in Leuk-1 cells. (F) qRT-PCR showed inhibition of TLR4 shRNA on LPS-induced (100 μg/mL) *IFITM4P* expression in Leuk-1 cells. (G) qRT-PCR showed inhibition of TAK-242 (1 μM) on LPS-induced (100 μg/mL) *IFITM4P* expression in Leuk-1 cells. (H) Inhibition of TAK-242 (1 μM) on IFITM4P promoter-driven luciferase activity in response to LPS (100 μg/mL) in 293T cells. (I) Schematic timeline of modified mouse tongue leukoplakia/SCC model. (J) (a) Normal tongue (cage I) and (b–d) typical tongue leukoplakia (cages II–IV). (c and d) Tongue leukoplakia lesions were larger and rougher in texture (cages III and IV). (e–h) Histopathological diagnoses: NM (e) (cage I), OL with moderate dysplasia (f) (cage II), OL with severe dysplasia and local early invasive SCC (g and h) (cages III and IV). (i–l) IHC Staining of PD-L1. Negative staining (i) (cage I). Staining of local early invasive SCC was stronger (k and l) (cages III and IV) than that of tongue leukoplakia (j) (cage II). Staining was stronger in early invasive SCC areas than in nearby OL areas (k). (m–p) FISH staining of IFITM4P. Negative staining (m) (cage I). Staining of local early invasive SCC was stronger (o and p) (cages III and IV) than that of tongue leukoplakia (n) (cage II). Staining was stronger in early invasive SCC areas than in nearby OL areas (o) (e–p, 200×). (K) qRT-PCR and WB confirmed that in 4NQO-induced tongue leukoplakia/SCC mouse model, the expression of IFITM4P increased with progression of the disease. Compared with the ddH_2_O control group, LPS significantly increased the expression of IFITM4P and promoted the carcinogenesis of tongue leukoplakia. (L) Schematic timeline of the PD-1 mAb treatment in early tongue leukoplakia mouse model. (M) PD-1 mAb is effective in leukoplakia treatment, especially for leukoplakia induced by 4NQO and LPS. Macroscopic view before and after PD-1 mAb treatment. (N) Ratio of tongue lesion score before and after PD-1 mAb treatment was evaluated. Data from (E), (F), and (G) are shown as mean ± SD from three independent experiments; ∗p < 0.05. Data from (B,C,D), (H), (J,K) and (M,N) are shown as mean ± SD from six independent experiments. ∗P < 0.05.

To determine the role of PD-L1 downregulation in IFITM4P-mediated cell proliferation, we stably transduced the vector (control) or IFITM4P in shPD-L1 and negative control (NC) Leuk-1/HN4 cells (Figures S2A and S2B). We assessed the effect of PD-L1 on cell growth using the cell counting kit-8 (CCK-8) assay. Knockdown of PD-L1 significantly inhibited the growth of the IFITM4P Leuk-1/HN4 cells to a level similar to that of the control Leuk-1 and HN4 cells (Figures S2C and S2D). To further clarify that IFITM4P is functionally involved in regulating the expression of PD-L1, we performed the T cell killing experiment. We demonstrated that after co-culture with T cells and shIFTM4P-Leuk1 and HN4 cells, cell death of knockdown of IFITM4P groups was much more than that of the NC group. Moreover, elevated PD-L1 in shIFTM4P decreased the cell death to a level comparable with that of the NC group (Figures S2E and S2F). Next, to test the influence of IFITM4P on anti-PD-1 therapy, we utilized an anti-PD-1 mAb to treat C57BL/6 mice inoculated with melanoma (B16F10) cells overexpressing the control vector or IFITM4P (Figure 4A). Tumor-bearing mice were treated with PD-1 mAb or an immunoglobulin G (IgG) isotype (IgG2a). The IFITM4P group showed a significantly higher tumor volume compared with the vector group (∗p < 0.05) (Figures 4B and 4C). Compared with the control IgG group, mice treated with PD-1 mAb showed a significant decrease in tumor volume (Figures 4B and 4C). Interestingly, the PD-1 mAb therapy had a significantly better tumor-inhibitory effect in the IFITM4P group than on the control group (∗p < 0.05) (Figures 4B and 4C). However, no significant difference was observed between the IFITM4P and vector groups in terms of mouse weight (Figure 4D).

Previous studies have found that inflammation in the oral cavity promotes OSCC progression^29^ via TLR4.^30^, ^31^, ^32^ To explore the role of TLR4 on the immune escape via IFITM4P during oral carcinogenesis, TLR4 ligand LPS was used as stimulus. First, we examined the effect of LPS on IFITM4P expression and found that LPS effectively induced IFITM4P transcription in a dose-dependent manner in Leuk-1 cells (Figures 4E and S3A). Polymyxin B (PmB) is often used to neutralize contaminated LPS by preventing the binding of LPS to TLRs.^33^ To investigate whether the active factor was LPS, we employed PmB to bind and inactivate LPS in the culture medium.^34^ Notably, the induction of IFITM4P by LPS was effectively abolished by the addition of PmB in Leuk-1 cells, while adding PmB alone to the cell did not affect the expression of IFITM4P (Figure S3B). We further validated the role of TLR4, which specifically recognized LPS^31^^,^^35^^,^^36^ in IFITM4P induction, using TLR4-specific shRNA in Leuk-1 cells and found that TLR4 deficiency inhibited LPS-induced expression of IFITM4P (Figures 4F and S4). Next, we evaluated the effects of TAK-242 (resatorvid), which is a small molecule that inhibits TLR4 signaling pathways and suppresses inflammatory reactions,^37^ on LPS-induced IFITM4P mRNA levels in Leuk-1 cells. LPS stimulation caused an increase in IFITM4P mRNA levels compared with controls. This response was ablated by the addition of TAK-242 (Figure 4G). These findings were confirmed by the luciferase reporter assay, which showed a significant increase in IFITM4P-associated luciferase activity following LPS stimulation, which was significantly decreased by TAK-242 treatment (Figure 4H).

To evaluate the effect of LPS-mediated IFITM4P on oral carcinogenesis in mice induced with 4NQO, we used a modified C57/B6J mouse tongue carcinogenesis model (Figure 4I). The results showed that the tongue leukoplakia in mice from cages III and IV were more severe, with leukoplakia on the dorsal tongue and local early invasive tongue SCC. PD-L1 and IFITM4P staining of tongue SCC from mice in cages III and IV were stronger than that of tongue leukoplakia from mice in cage II, while the normal tongue mucosa from mice in cage I was not stained (Figure 4J). Consistently, WB and qRT-PCR analysis showed that the expression of IFITM4P and PD-L1 was similar to that of IHC and FISH staining (Figure 4K). Furthermore, to verify the influence of IFITM4P on anti-PD-1 therapy, we utilized PD-1 mAb to treat mouse early tongue leukoplakia induced with 4NQO + LPS or 4NQO alone (Figure 4L). The results showed that mouse tongue leukoplakia was significantly relieved after 12 days of PD-1 mAb treatment. Moreover, compared with the 4NQO induction group, PD-1 mAb was more effective in the 4NQO + LPS induction group. (Figures 4M and 4N). Taken together, these results demonstrated that LPS accelerated tongue carcinogenesis in the mouse model, elevated IFITM4P expression, and induced a tumor-immunosuppressive effect through PD-L1 upregulation. Moreover, elevated IFITM4P expression increased the therapeutic sensitivity of PD-1 mAb therapy. High IFITM4P might, therefore, be an indicator of PD-1 mAb therapeutic sensitivity during oral carcinogenesis.

### Cytoplasmic IFITM4P interacts with the SH3 domain of SASH1 to promote *PD-L1* transcription through the TAK-1/NF-κB pathway in OL

**Figure 5:**
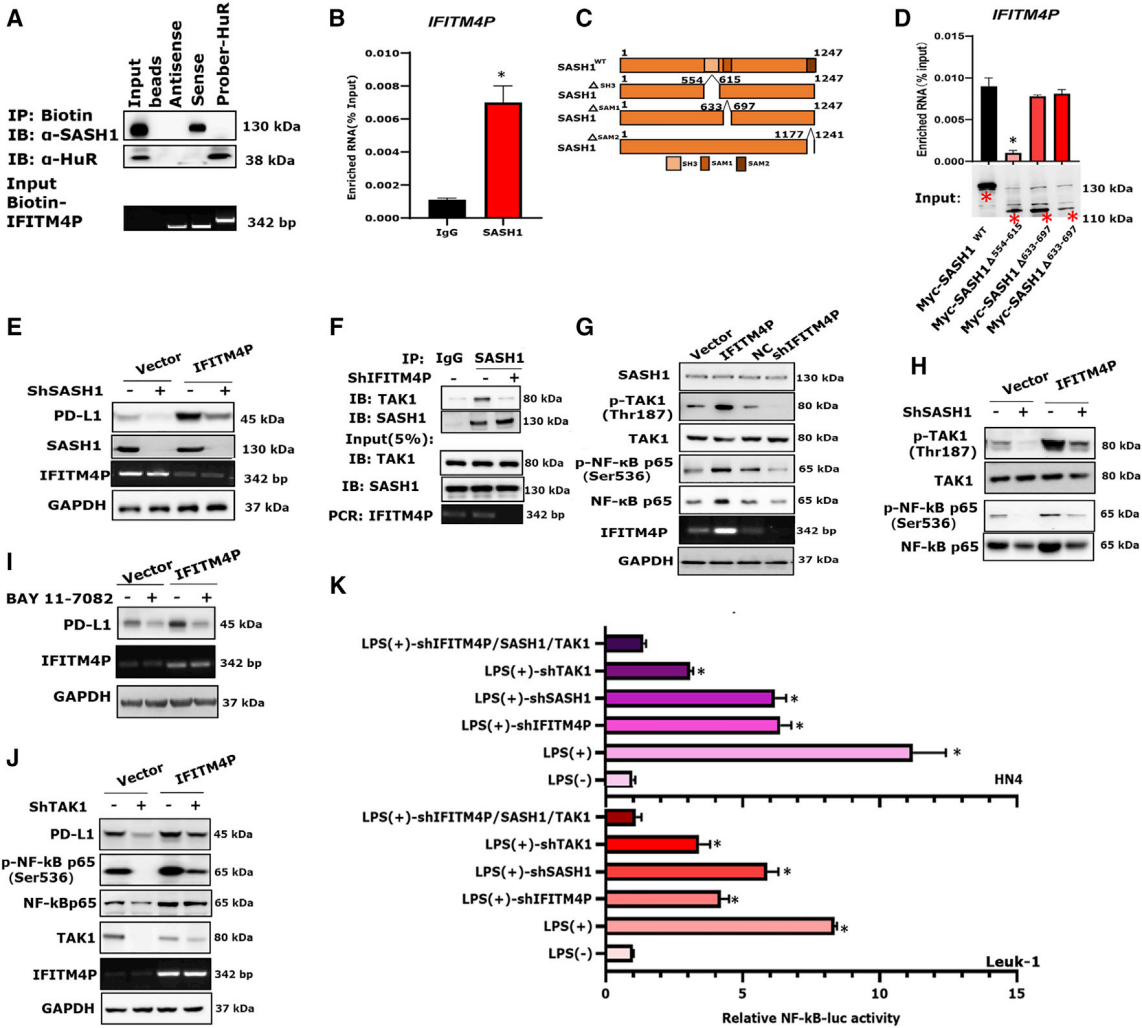
Interaction of IFITM4P and SASH1 promoted the expression of PD-L1 through the TAK1-NF-κB signaling pathway (A) Biotin-labeled IFITM4P pull-down and WB showed that IFITM4P specifically co-precipitates with SASH1 in Leuk-1 cells. Biotin-HuR and antisense served as positive and negative controls, respectively. (B) RIP assays validated the association of SASH1 with IFITM4P in Leuk-1 cells. Antibodies against GAPDH or control IgG served as controls. (C and D) Different truncated forms of SASH1 (C) and their binding to IFITM4P using RIP assays in Leuk-1 cells (D). (E) WB showed that the expression of PD-L1 in IFITM4P overexpressing cells was significantly decreased upon knockdown of SASH1 using ShSASH1. (F) Detection of the endogenous interaction between IFITM4P, TAK1, and SASH1 in Leuk-1 cells by co-IP and qRT-PCR analysis. (G) WB analysis showed that pTAK1 (Thr187) and pNF-κB p65 (Ser536) expression were increased following the ectopic expression of IFITM4P in Leuk-1 cells and decreased by knockdown of IFITM4P. (H) WB analysis showed a decrease in the levels of NF-κB, pNF-κB (Ser536), and pTAK1 (Thr187) following depletion of SASH1 in control and IFITM4P-expressing Leuk-1 cells. (I) WB analysis showed a decrease in PD-L1 expression in control and IFITM4P expressing cells treated with BAY 11-7082 (10 μM). (J) WB analysis showed that knockdown of TAK1 with shTAK1 repressed *PD-L1* transcription in IFITM4P expressing Leuk-1 cells. (K) NF-κB-driven luciferase activity was enhanced in HN4 and Leuk-1 cells stimulated with LPS. Transfection of these cells with shTAK1, shSASH1, or shIFITM4P led to an inhibition of LPS-induced NF-κB signaling. pGL3.0 was used as a control. Data from (B) and (D) are shown as mean ± SD from three independent experiments; ∗p < 0.05. Data from (K) are shown as mean ± SD from six independent experiments; ∗p < 0.05.

We performed biotin-labeled RNA pull-down assays followed by mass spectrometry (MS) analyses to identify proteins interacting with IFITM4P. To reduce the non-specificity of the RNA pull-down/MS results, we used FISH to locate the expression of IFITM4P in Leuk-1 cells. Interestingly, FISH staining showed that the expression of IFITM4P was predominantly localized in the cytoplasm (Figure S5). SAM and SH3 domain containing protein 1 (SASH1) is a scaffold protein in TLR4 signaling for assembly of a signaling complex downstream of TLR4 to activate early endothelial responses to receptor activation.^38^ Notably, we identified SASH1 as a potential interacting protein of IFITM4P. Furthermore, we performed biotin-labeled IFITM4P pull-down and verified its interaction with SASH1 by WB, using biotin-HuR as a positive control (Figure 5A). RNA immunoprecipitation (RIP) assays showed that SASH1 could significantly enrich IFITM4P compared with the controls (Figures 5B and S6).

SASH1 protein can be divided into the SH3 (amino acids 554–615), SAM1 (amino acids 633–697), and SAM2 (amino acids 1,177–1,241) domains (Figure 5C). To identify the domain that mediated the interaction with IFITM4P, we first generated a 554- to 615-Myc-tagged truncation of SASH1 and performed RIP assays with Myc antibody. Notably, the full-length protein, as well as the SAM1 and SAM2 domains of SASH1, were bound to IFITM4P, whereas the SH3 domain was not (Figure 5D), suggesting that the amino acid residues 554–615 are critical for SASH1 binding. Knockdown of SASH1 resulted in repression of *PD-L1* expression (Figures 5E and S7). Moreover, the expression of PD-L1 in IFITM4P-overexpressing cells also showed a significant decrease upon knockdown of SASH1 (Figure 5E). We next analyzed the interaction network of SASH1-associated proteins and TAK1 (MAP3K7) as potential interactors of SASH1 (https://www.genecards.org/) (Figure S8).

To determine whether there is a physical interaction between SASH1 and TAK1 and whether it depends on IFITM4P, we transiently expressed Sh-IFITM4P and performed co-immunoprecipitation (co-IP) assays using TAK1. SASH1 did not bind TAK1 when IFITM4P was knocked down in Leuk-1 cells (Figure 5F). To further understand the mechanism of IFITM4P-mediated PD-L1 regulation, we assessed the mRNA levels of TAK1 and SASH1 following the overexpression and knockdown of IFITM4P, respectively. The results showed that IFITM4P did not have any apparent effect on TAK1 and SASH1 (Figures S9A and S9B). We next examined the phosphorylation status of TAK1 (Thr187) and TAK1 (Thr412) by WB with increasing expression of IFITM4P (Figure S9C). While phosphorylation of TAK1 at Thr187 showed a positive correlation with IFITM4P expression, phosphorylation of TAK1 at Thr412 showed no apparent change (Figure S9C). Moreover, depletion of IFITM4P resulted in decreased TAK1 (Thr187) phosphorylation, while the elevation of IFITM4P increased its phosphorylation (Figure 5G). TAK1 is involved in nuclear factor κB (NF-κB) activation. GSEA showed that the hallmarks of malignant tumors, including NF-κB signaling, were significantly enriched in Leuk-1 cells with risk scores higher than the median risk score (Figure S1A). Consistent with these findings, we found that the stable expression of IFITM4P increased NF-κB (Ser536) phosphorylation, while its knockdown decreased NF-κB (Ser536) phosphorylation (Figure 5G). Moreover, depletion of SASH1 resulted in decreased expression of NF-κB, p-NF-κB (Ser536), and p-TAK1 (Thr187) in both vector and IFITM4P expressing Leuk-1 cells (Figures 5H and S9D). Interestingly, the association of NF-κB was also reduced in shIFITM4P-Leuk-1 cells and vice versa (Figures 5G, 5H, and S10A). BAY 11-7082 is an inhibitor of IκBα phosphorylation, which stabilizes IκBα and specifically blocks the NF-κB signaling pathway.^39^ Treatment with BAY 11-7082 led to a decrease in PD-L1 expression in both vector and IFITM4P expressing cells (Figures 5I and S10B). Furthermore, knockdown of TAK1 resulted in decreased levels of PD-L1 in IFITM4P-Leuk-1cells (Figures 5J and S10C). NF-κB-driven luciferase activity was enhanced in HN4, and Leuk-1 cells were stimulated with LPS. However, transfection of these cells with shTAK1, shSASH1, or shIFITM4P led to an inhibition of LPS-induced NF-κB signaling, indicating that the NF-κB activity was completely dependent on the TAK1/SASH1/IFITM4P expression (Figure 5K). Together, these data indicate that IFITM4P promotes immune evasion in OL by regulating the SASH1-TAK1-NF-κB-PD-L1 axis.

### LPS partially induces the entry of IFITM4P into the nucleus, enhances the binding of KDM5A to the *Pten* promoter, and reduces *Pten* transcription

**Figure 6:**
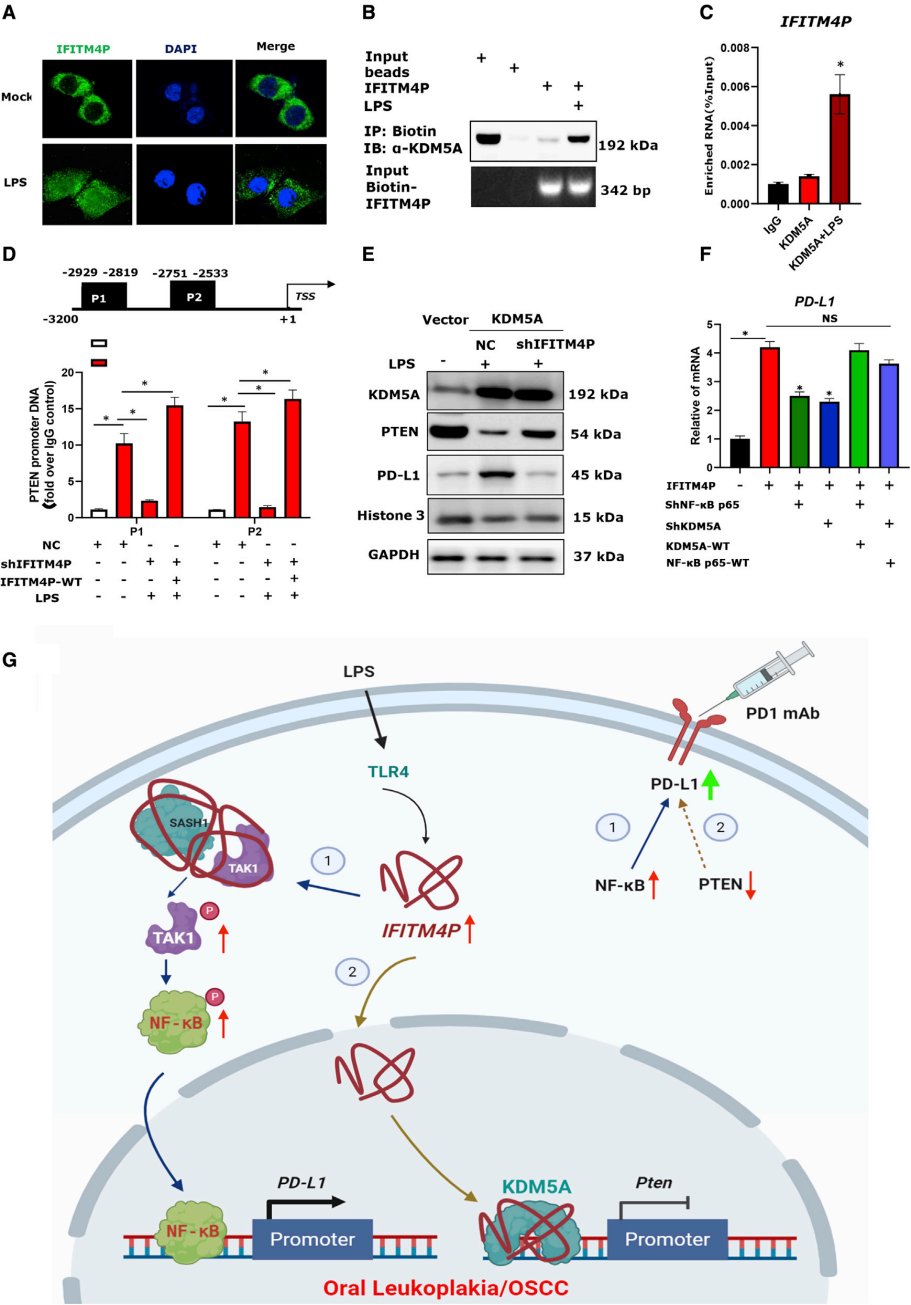
Ectopic expression of IFITM4P effectively enhanced the binding of KDM5A to Pten promoter and increased PD-L1 abundance (A) Confocal microscopy showed IFITM4P was obviously transferred from the cytoplasm to the nucleus in Leuk-1 under LPS stimulation (100 μg/mL) by FISH assays (400×). Assessment of the nuclear morphology used DAPI staining. (B) Biotin-labeled IFITM4P pull-down and WB showed that IFITM4P specifically co-precipitates with KDM5A in Leuk-1 cells upon LPS stimulation (100 μg/mL) for 12 h. Beads served as the negative control. (C) RIP assays validated the association of KDM5A with IFITM4P in Leuk-1 cells upon LPS stimulation (100 μg/mL) for 12 h. IgG antibodies served as the control. (D) ChIP analysis of the Pten promoter in Leuk-1 cells. Upper: KDM5A binding sites. Lower: ChIP assays showed that KDM5A specifically bound P1 and P2. Deletion of IFITM4P alone markedly reduced the binding of KDM5A to Pten, while transfecting the plasmid of IFITM4MP-WT in stable knockdown-IFITM4P-Leuk-1 can reduce the inhibitory effect on the binding of KDM5A to Pten. ChIP assays were performed with anti-KDM5A antibody or IgG as a control. Enriched DNA fragments flanking P1 and P2 were examined by RT-PCR assays using specific primer sets. (E) WB analysis of relative *Pten* mRNA expression in Vector and KDM5A-overexpressing Leuk-1 cells under LPS stimulation (100 μg/mL). (F) IFITM4P-Leuk-1 and Vector-Leuk-1 cells transiently transfected with shRNAs to NF-κB p65 or KDM5A or scrambled control (ShRNA-NC), KDM5A, or NF-κB p65 vector were treated with LPS (100 μg/mL). qRT-PCR showed a significant decrease in PD-L1 expression following the knockdown of KDM5A and NF-κB p65, while WT-KDM5A or WT-NF-κB p65 increased the expression of *PD-L1*. (G) Model describing the role of IFITM4P as an oncogene by increasing PD-L1 abundance in OL. Data from (C), (D), and (F) are shown as mean ± SD from three independent experiments; ∗p < 0.05; NS, no significant difference. WT, wild type.

Interestingly, in the previous study we first performed FISH assays to detect the subcellular localization of IFITM4P upon stimulation by LPS. Strikingly, IFITM4P was obviously transferred from the cytoplasm to the nucleus in Leuk-1 under LPS stimulation (Figure 6A). A previous study^40^ showed that increased amounts of histone 3 lysine 4 demethylase KDM5A in tumors markedly improved the response to treatment with PD-1 antibody in mouse cancer models. According to our result, biotin-labeled RNA pull-down followed by MS analyses confirmed the interaction of IFITM4P with KDM5A (Figures S11A and S11B). Furthermore, we performed biotin-labeled IFITM4P pull-down and verified its interaction with KDM5A by WB using biotin-HuR as a positive control. The results indicate that the interaction between IFITM4P and KDM5A depends on LPS stimulation (Figure 6B). Furthermore, RIP assays showed that KDM5A could significantly enrich IFITM4P compared with controls with LPS (Figure 6C). KDM5A increased the abundance of PD-L1 in tumor cells by suppressing the PTEN expression pathway and inducing PI3K-AKT-S6K signaling, and directly interacted with the *Pten* promoter (∼3 kb proximal to the transcription start site) to repress *Pten* transcription.^40^ We performed a chromatin immunoprecipitation (ChIP) assay to determine whether IFITM4P regulates the binding of KDM5A to the *Pten* promoter. The results showed that KDM5A was preferentially bound to the P1 (−2,929 to −2,819) and P2 (−2,751 to −2,533). Deletion of IFITM4P alone markedly reduced the binding of KDM5A to *Pten*, while transfecting the plasmid with IFITM4MP-WT (wild type) in stable knockdown-IFITM4P-Leuk-1 can reduce the inhibitory effect on the binding of KDM5A to Pten (Figure 6D). We also used qPCR and WB to confirm that overexpression of KDM5A reduces the abundance of PTEN at the transcript and protein levels, and knocked down IFITM4P can reduce this inhibition (Figures 6E and S12). Furthermore, qRT-PCR showed a significant decrease in PD-L1 expression following the knockdown of KDM5A and NF-κB, while KDM5A or NF-κB increased the expression of PD-L1 in Leuk-1 (Figure 6E) and HN4 cells (Figure S13). To explore the clinical correlation between IFITM4P and PTEN, TAK1, SASH1, and NF-κB, we analyzed their expression in HNSC (n = 519) samples found in TCGA. Notably, IFITM4P expression was negatively associated with that of PTEN (p = 0.001, r = −0.14, n = 519; Figure S14), but there was no correlation between other genes. Together, these data indicate that LPS partially induces the entry of IFITM4P into the nucleus, enhances the binding of KDM5A to the *Pten* promoter, and reduces *Pten* transcription, thereby upregulating PD-L1 in OL.

## Discussion

High-throughput detection of cancer genomes and epigenomes has defined large numbers of driver mutations and molecular subgroups, leading to therapeutic advances. However, there is a relative paucity of such knowledge in premalignancy, which inherently limits the potential to develop precision prevention strategies.^41^ In the present study an lncRNA, IFITM4P, which was activated through LPS/TLR4 and upregulated PD-L1 via dual mechanism during oral carcinogenesis, was reported. IFITM4P, transcribed from interferon-induced transmembrane protein 4 pseudogene (IFITM4P), was reported a few months ago^42^^,^^43^ for its function in regulating host antiviral responses.

Protein can shuttle between the nucleus and the cytoplasm. Whereas many proteins are selectively transported from the cytoplasm into the nucleus, most RNAs are exported from the nucleus to the cytoplasm. In contrast to mRNAs, which function in the cytoplasm, small nuclear RNAs (snRNAs) function within the nucleus as components of the RNA processing machinery. Furthermore, snRNAs can shuttle between the nucleus and the cytoplasm. Until now, there has been no evidence that lncRNAs shuttle between the nucleus and the cytoplasm. In the present study, we first report a novel nucleocytoplasmic shuttle gene, IFITM4P, as a bona fide activator to promote immune escape in OL cells. Furthermore, IFITM4P acts as a scaffold to facilitate recruitment of SASH1 to bind and phosphorylate TAK1 (Thr187) and further increase the phosphorylation of NF-κB (Ser536) to directly induce PD-L1 transcription, thus activating an immunosuppressive program that allows OL and OSCC cells to escape anti-cancer immunity in the cytoplasm.

According to our result, in the nucleus, IFITM4P enhances the binding of KDM5A to the *Pten* promoter and reduces *Pten* transcription, thereby upregulating PD-L1 in OL cells. KDM5A may have multiple mechanisms of promoting PD-L1 abundance. Studies have shown that KDM5A increases the abundance of PD-L1 in tumor cells by inhibiting PTEN expression and inducing PI3K-AKT-S6K signal transduction.^40^ Our research found that LPS can induce IFITM4P to partially enter the nucleus, enhance the binding of KDM5A to the Pten promoter, and reduce Pten transcription, thereby upregulating PD-L1 in OL cells.

SASH1 is a large protein with a predicted molecular mass of 137 kDa that belongs to the SAM and SH3 adapter family proteins. It consists of the SH3 domain expressed in lymphocytes (SLY1) and a hematopoietic adapter containing SH3 and SAM domain 1 (HACS1, also called SLY2).^38^^,^^44^ Activation and cleavage of SASH1 by caspase-3 has been shown to mediate an apoptotic response that is NF-κB dependent. These findings revealed that SASH1 might be an adapter protein for multiple signaling pathways. We found that the IFITM4P/SASH1 complex acted as a scaffold molecule by binding to the TAK1 complex, leading to NF-κB p65 activation in OL and OSCC cells. NF-κB was verified to be a key positive regulator of PD-L1 expression in many kinds of cancer. This process was largely dysregulated in cancer. Upregulation of PD-L1 in cancer cells was controlled via NF-κB downstream of several signals, including oncogene- and stress-induced pathways, and inflammatory cytokines.^45^ TLR4 was verified to protect the tumor from immune attack in HNSC/OSCC.^30^, ^31^, ^32^ In the present study, TLR4 ligand LPS^35^ was shown to promote the carcinogenesis of mouse tongue leukoplakia by increasing IFITM4P and PD-L1 levels. Our results also showed that IFITM4P is dramatically induced by the LPS/TLR4 pathway during oral carcinogenesis.

To evaluate the effect of LPS/TLR4-mediated IFITM4P during oral carcinogenesis *in vivo*, we used a modified C57/B6J mouse tongue carcinogenesis model. We found that LPS accelerated the development of tongue SCC from tongue leukoplakia *in vivo*, elevated IFITM4P expression, induced a tumor-immunosuppressive effect through PD-L1 upregulation, and increased the therapeutic sensitivity of PD-1 mAb therapy. However, so far there is no clinical trial report on the effectiveness PD-1 mAb in OL treatment. To carry out more relevant clinical trials for blockage of OL malignant transformation, a deeper understanding of the molecular characteristics of oral carcinogenesis is urgent.

In summary, IFITM4P is progressively induced from OL to OSCC cells through the LPS/TLR4 pathway, and high expression of IFITM4P results in increased OSCC cell proliferation and enhanced immune escape via induction of *PD-L1* expression. Mechanistically, IFITM4P induces *PD-L1* via a dual pathway: in the cytoplasm IFITM4P acts as a scaffold to facilitate recruitment of SASH1 to bind and phosphorylate TAK1 (Thr187) and further increase the phosphorylation of NF-κB (Ser536) to induce PD-L1 transcription; and in the nucleus IFITM4P reduced *Pten* transcription by enhancing the binding of KDM5A to the *Pten* promoter, thereby upregulating PD-L1 in OL cells. Moreover, mice bearing tumors with high IFITM4P expression exhibit notably therapeutic sensitivity to PD-1 mAb treatment. Collectively, these data demonstrate that IFITM4P may serve as a new therapeutic target in the blockage of oral carcinogenesis, and PD-1 mAb can be an effective reagent to treat OSCC with high expression of IFITM4P.

## Materials and methods

### OL/OSCC patients and specimens

The tissue samples for lncRNA microarray experiments were obtained from a stepwise setting (Table S1) from human oral NM (n = 3) to OL (n = 4) to OSCC (n = 5). The tissue samples for qRT-PCR validation were obtained from an independent setting (Table S2), including NM (n = 23), OL (n = 67), and OSCC (n = 46). This study was approved by the Institutional Review Board of the Shanghai Ninth People’s Hospital (approval number: SH9H-2016-80-T37). All procedures performed in studies involving human participants were in accordance with the ethical standards of the institutional and/or national research committee and with the 1964 Helsinki declaration and its later amendments or comparable ethical standards. The histologic examination of all subjects was performed by two oral pathologists from the department of oral pathology in our hospital and was based on the WHO criteria.^46^ All patients diagnosed with primary OL or OSCC were not treated before biopsy or surgery.

### Cell culture and drugs

Leuk-1 cells were cultured in keratinocyte serum-free medium (cat. no.10744, Gibco, Waltham, MA, USA), and HN4 cells were cultured in DMEM supplemented with 10% fetal bovine serum. Information on the drugs used is presented in Table S3.

### lncRNA microarray analysis, RNA-seq, and qRT-PCR validation

Total RNA was extracted from cultured cells and tissue samples using TRIzol reagent according to the manufacturer's protocol (TaKaRa, Dalian, Japan). For microarray analysis, the Affymetrix (Santa Clara, CA, USA) Gene ChipR Human Transcriptome Array 2.0 was employed according to the manufacturer's protocol.

RNA-seq of samples was performed on an Illumina HiSeq X Ten sequencing system and was detected by Novogene (Liebing Bioinformatics Technology, China).

For expression validation, the PrimeScript RT reagent kit with gDNA Eraser (TaKaRa) was used to synthesize cDNA. The expression levels of mRNA were detected using RT-PCR with SYBR green (TaKaRa) according to the manufacturer's instructions. The primers used for qRT-PCR are presented in Table S4.

### Immunohistochemistry and RNAscope

PD-L1-IHC staining was performed as previously described.^47^ OL and OSCC slides were probed for IFITM4P expression using the RNAscope Red Manual Assay (Advanced Cell Diagnostics, Newark, CA, USA) per the manufacturer's recommendations. The probes used were IFITM4P, hs-PPIB-1ZZ (positive control, 701041), and dapB (negative control, 701021) (Advanced Cell Diagnostics).

### Cell viability and colony formation assays

Cell viability was assessed via an enzyme-linked immunosorbent assay by plating cells (1 × 10^4^ per well) in 96-well plates. Cell proliferation reagent CCK-8 was performed as previously described.^47^^,^^48^ The absorbance was measured at 480 nm against a background control using a microplate reader.

IFITM4P-overexpressing in Leuk-1 and HN4 cells, and IFITM4P-knockdown cells and their corresponding control cells, were seeded (1 × 10^3^) into 12-well plates for 14 days, and colonies of more than 50 cells were counted under a dissecting microscope.

### Co-immunoprecipitation, RNA pull-down, and RNA immunoprecipitation assays

Detailed methods for co-IP have been described previously.^49^^,^^50^ The IFITM4P-binding proteins were studied by RNA pull-down assays using the Pierce Magnetic RNA-Protein Pull-Down Kit (Thermo Fisher Scientific, Waltham, MA, USA) according to the manufacturer's instructions. Biotinylated IFITM4P and antisense sequences were synthesized using a TranscriptAid T7 High Yield Transcription Kit (Thermo Fisher Scientific). The cytoplasmic fraction obtained using an NE-PER Protein Extraction Kit (Thermo Fisher Scientific) was incubated overnight with biotinylated with IFITM4P, followed by precipitation with streptavidin magnetic beads. The retrieved protein was eluted from the RNA-protein complex and analyzed by immunoblotting or silver staining. Silver staining was performed using a silver staining kit (Beyotime, China) according to the manufacturer's instructions. Ubiquitinating sites were studied via MS (Novogene Bioinformatics Technology, Beijing, China).

The RIP assays were performed using an EZ-Magna RIP kit (Millipore). Leuk-1 cells (4 × 10^8^) were lysed using a complete RIP lysis buffer. The lysates were immunoprecipitated in RIP buffer with anti-HuR antibody-conjugated magnetic beads (Abcam, Cambridge, UK), SASH1 antibody, or IgG. The precipitated RNAs were analyzed by qRT-PCR. Mouse IgG and HuR RNA were used as negative and positive controls, respectively.

### Chromatin immunoprecipitation assay

ChIP assays were conducted with a ChIP Assay Kit (Cell Signaling Technology [CST], Danvers, MA, USA). In brief, Leuk-1 cells (5 × 10^8^) were fixed with a final concentration of 1% formaldehyde, crosslinked, and sonicated. The KDM5A antibody (10 μg/mL, Abcam), IgG control antibody (2 μg/mL, Abcam) was added to sonicated lysates and incubated overnight at 4°C, then incubated with a Protein A/G beads mixture (1:1 ratio, CST) for another >7 h at 4°C. Chromatin DNA was eluted, reverse crosslinked, and recovered using a ChIP Assay Kit (CST). Input DNA and immunoprecipitated DNA were analyzed by qPCR using promoter DNA-specific primers listed in Table S4.

### Modified mouse tongue leukoplakia/SCC model treated with PD-1 mAb therapy

Eight-week-old male C57Bl/6J mice, purchased from Shanghai Lingchang Biotechnology Science and Technology (China), were used. The carcinogen 4NQO (Sigma, St. Louis, MO, USA) solution was prepared in double-distilled water (ddH_2_O) at 100 μg/mL with overnight stirring at room temperature 1 day before the drinking water was changed. LPS (Solarbio, Beijing, China) solution was prepared in ddH_2_O at 10 μg/mL on the day drinking water was changed. A total of 20 mice were assigned into four cages (I–IV) of 5 mice per cage. The drinking plan and PD-1 mAb treatment method for each cage are shown in Figures 4I and 4L.

To test the effect of IFITM4P on anti-PD-1 therapy, we used another *in vivo* mouse model^51^ in this study. For the immune-competent mouse model, B16F10 cells (5 × 10^5^ cells in 100 μL of medium) expressing IFITM4P or the empty vector were injected subcutaneously into C57BL/6 mice. Tumor growth was measured using digital calipers, and tumor sizes were recorded. All animal experiments were approved by the Institutional Animal Care and Use Committee of Shanghai in accordance with the National Research Council Guide for Care and Use of Laboratory Animals (SCXK [Shanghai 2007–0005]).

### Data resources and analysis

The TCGA HNSC tumor tissues and paired normal tissue samples' RNA-seq data and corresponding clinical data were downloaded from https://portal.gdc.cancer.gov/projects/TCGA-HNSC.

Detailed analysis methods have been described previously.^52^ A p value of <0.05 was considered statistically significant. Statistical analysis was performed using SPSS 18.0 (SPSS, Chicago, IL, USA).

### Statistical analysis

Data, shown as mean ± standard deviation (SD), were analyzed using the independent Student's t test. The correlation between the expression of IFITM4P and PD-L1 in OL and OSCC was analyzed using Spearman’s rank correlation coefficient test. A p values of <0.05 were considered significant. Statistical analysis was performed using SPSS 18.0.

### Availability of data and materials

RNA-seq data from this study have been submitted to the Gene Expression Omnibus under the accession number GEO: GSE167292.

## Supplemental information

Document S1. Figures S1–S14 and Tables S1–S4

Document S2. Article plus supplemental information
