# Supplementary material for: LncRNA IFITM4P promotes immune escape by up-regulating PD-L1 via dual mechanism in oral carcinogenesis
Source: Mol Ther. 2022 Jan 17;30(4):1564–77. doi: 10.1016/j.ymthe.2022.01.003 (PMC9077312; doi:10.1016/j.ymthe.2022.01.003)
Supplement: Document S1. Figures S1–S14 and Tables S1–S4 [file mmc1.pdf]

YMTHE, Volume *30*

Supplemental Information

LncRNA IFITM4P promotes immune escape by up-regulating PD-L1 via dual mechanism in oral carcinogenesis

Linjun Shi, Yuquan Yang, Mengying Li, Chenxi Li, Zengtong Zhou, Guoyao Tang, Lan Wu, Yilin Yao, Xuemin Shen, Zhaoyuan Hou, and Hao Jia

Supplementary Figure. 1

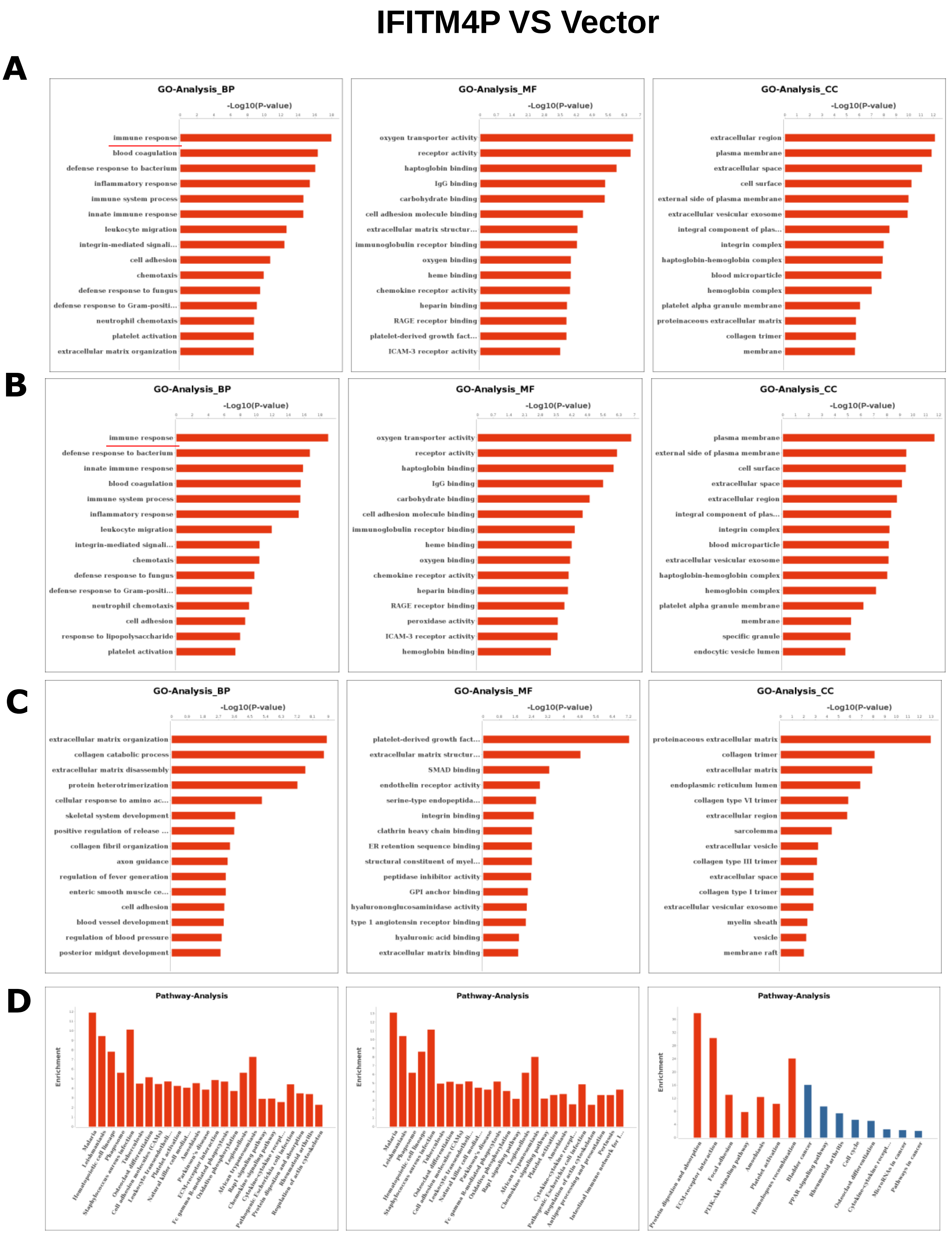

Enriched GO functions and KEGG pathway-Analysis of differentially expressed genes. GO analysis of all changed genes (A), upregulated (B)and down-regulated (C) genes.

(D) KEGG pathway analysis of differentially expressed genes. Left: All of changed genes KEGG pathway analysis,middle: upregulated genes, right: downregulated genes.

KEGG pathway analysis. KEGG, Kyoto Encyclopedia of Genes and Genomes.

Supplementary Figure. 2

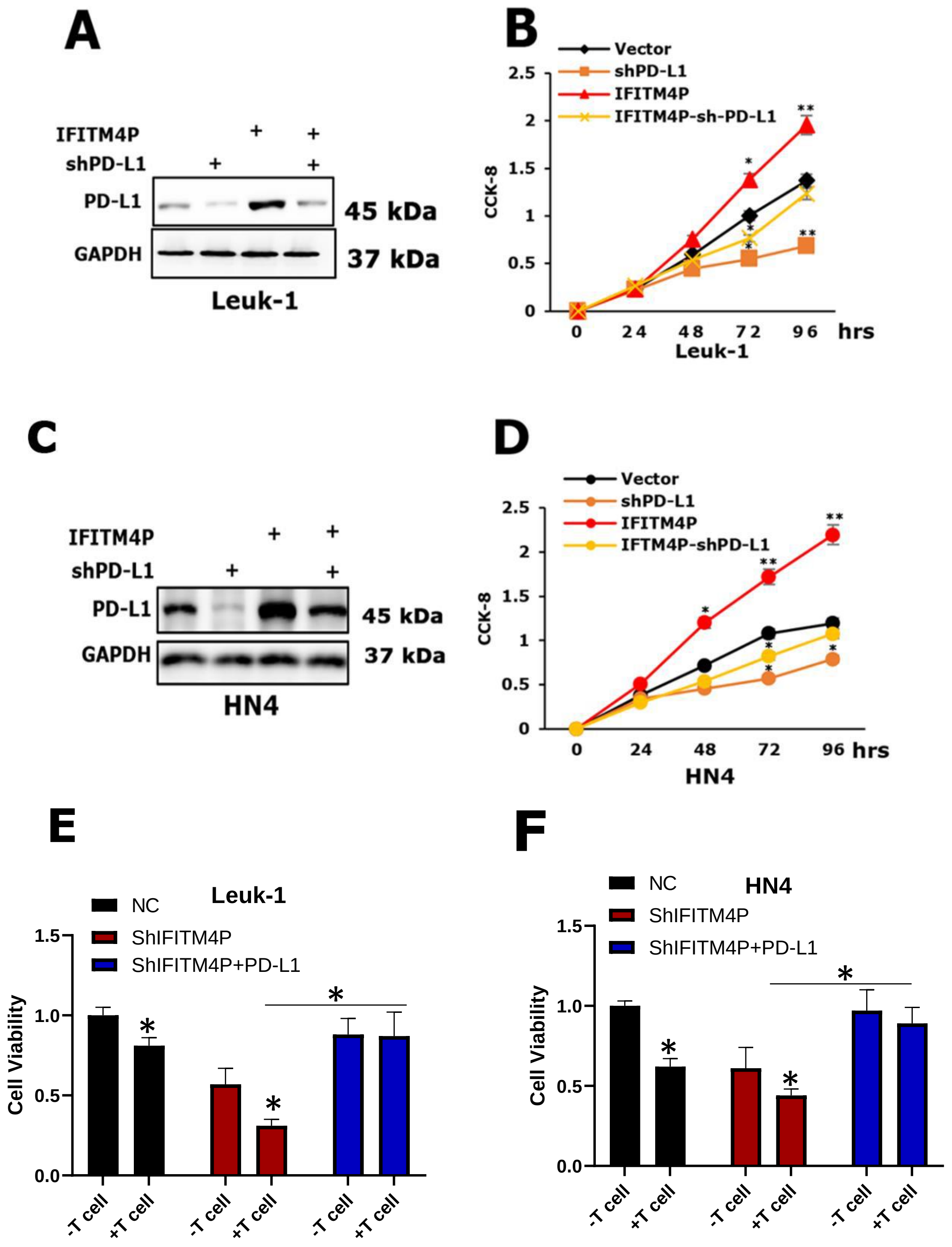

(A)Stable expression of PD-L1 treated with IFITM4P and shPD-L1 in Leuk-1 cells.
(B)Effect of IFITM4P and shPD-L1 on growth in Leuk-1 cells overexpressing the empty vector or IFITM4P-Leuk-1. Cell proliferation was measured using the CCK-8 assay.
(C)Stable expression of PD-L1 in HN4 cells treated with IFITM4P and shPD-L1.
(D)Effect of IFITM4P and shPD-L1 on growth in HN4 cells overexpressing the empty vector or IFITM4P-Leuk-1. Cell proliferation was measured using the CCK assay.
(E,F)T cell-mediated cancer cell-killing assay results. Leuk-1(E) or HN4(F) cells cocultured with activated T cell for 48 h were subjected to crystal violet staining. The ratio of Leuk-1 or HN4 to T cell is 1:3.

Supplementary Figure. 3

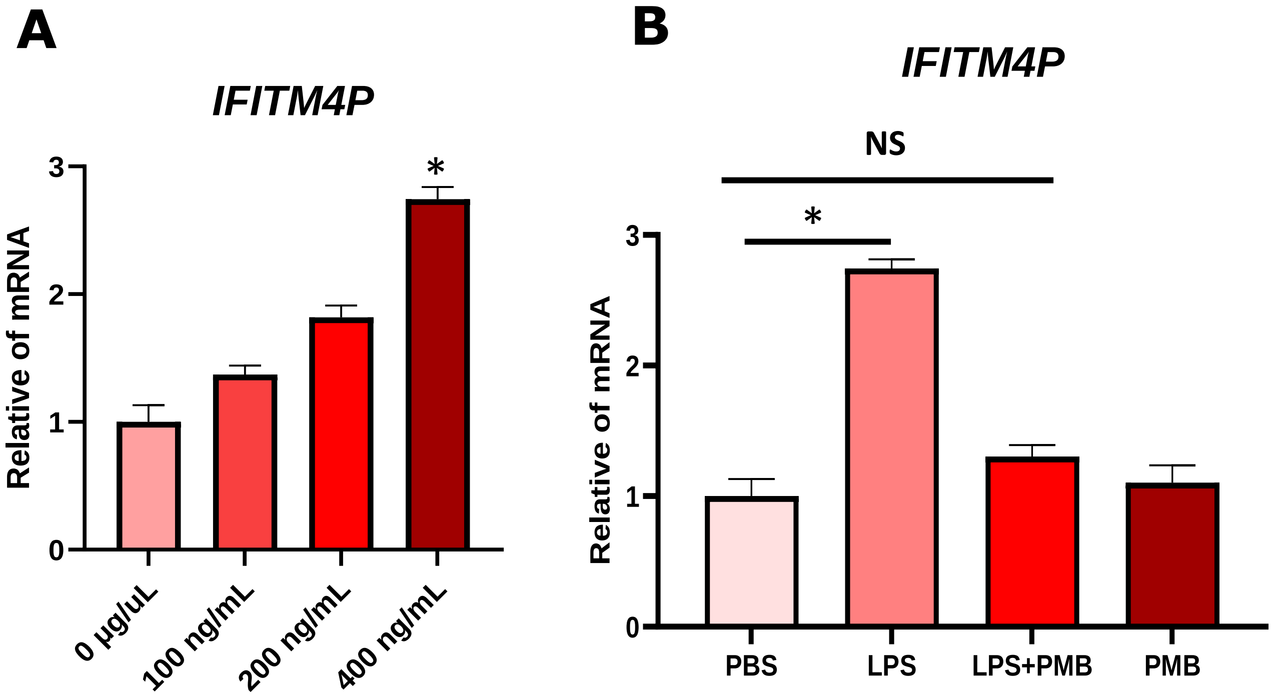

(A) qRT-PCR showed a dose-dependent increase in LPS-induced *IFITM4P* transcription after 12hrs in Leuk-1 cells.

(B) Comparison of the effects of LPS (400 ng/mL) alone and LPS (400 ng/mL) +PMB (10 μg/mL) treatment on *IFITM4P* after 12 hrs of incubation. Data were shown as mean ± s.d. from three independent. NS = no significant difference. *P < 0.05.

Supplementary Figure. 4

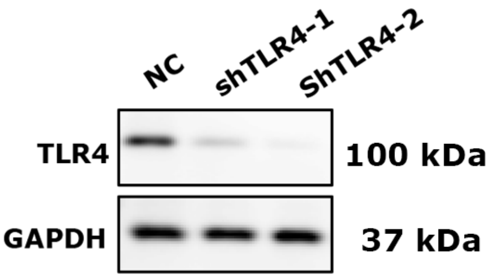

Western blotting shows stable expression of shTLR4 in Leuk-1 cells.

Supplementary Figure. 5

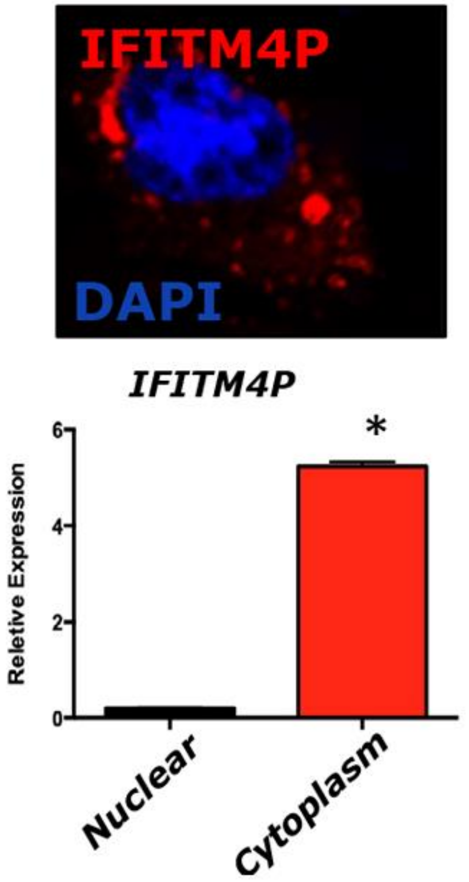

Top: Images taken by confocal microscopy shows the localization of IFITM4P detected by FISH assays in Leuk-1 cells. A representative image is shown at 400× magnification.

Bottom: qRT-PCR shows IFITM4P expression in the nuclear and cytoplasmic fractions of Leuk-1 cells. Data were shown as mean ± s.d. from three independent. *P < 0.05.

Supplementary Figure. 6

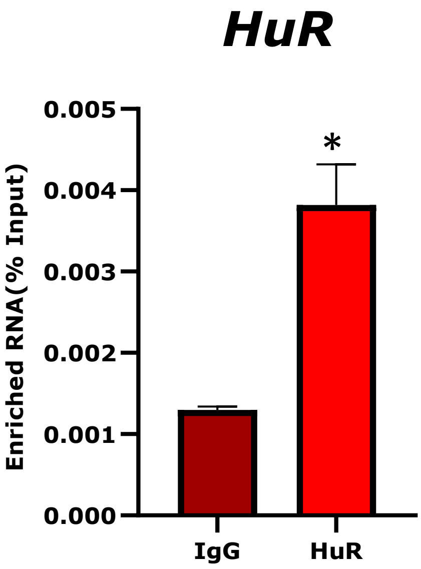

RIP assays validate the association of HuR-prober with HuR in Leuk-1 cells. HuR served as positive controls. (n=3), Data were shown as mean ± s.d. from three independent. *P < 0.05.

Supplementary Figure. 7

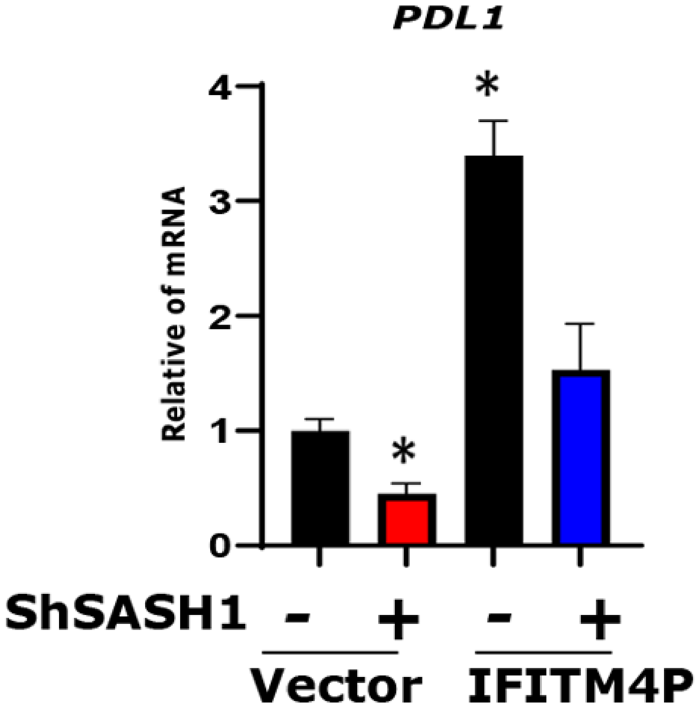

qRT-PCR show that the expression of *PD-L1* in IFITM4P overexpressing cells is significantly decreased upon knockdown of SASH1 using ShSASH1. Data were shown as mean ± s.d. from three independent. *P < 0.05.

Supplementary Figure. 8

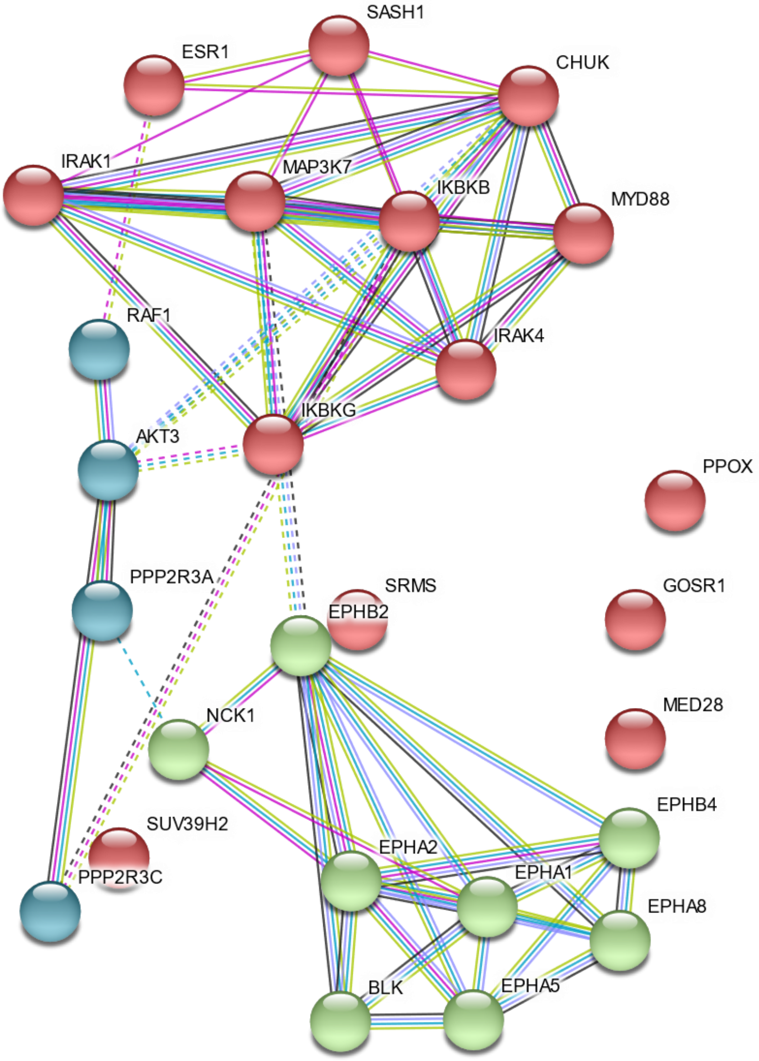

Bioinformatics analysis of the interaction network of SASH1 associated proteins (https://www.genecards.org/). MAP3K7 (TAK1)

Supplementary Figure. 9

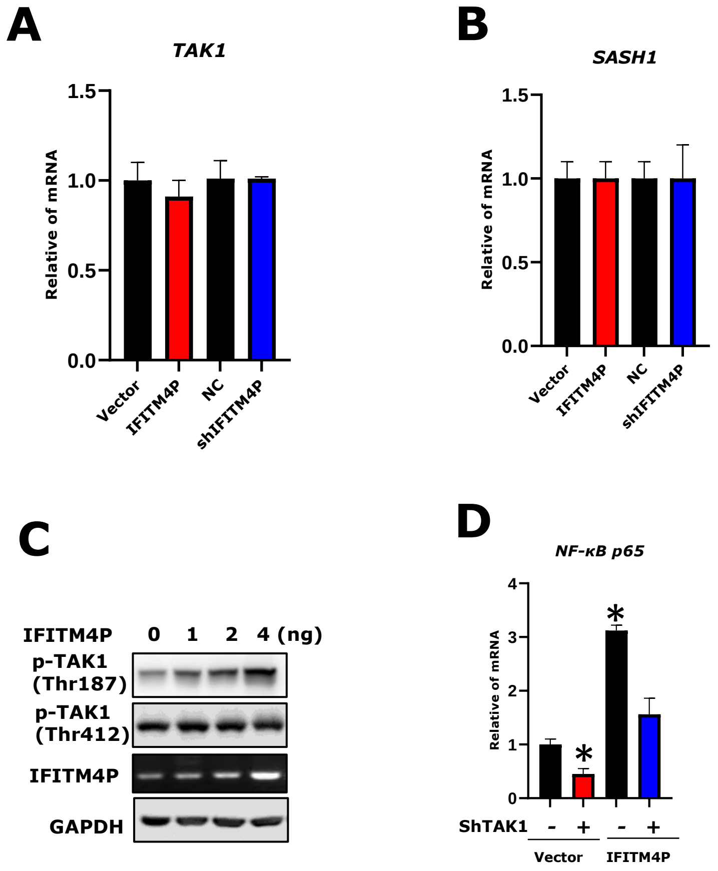

(A) (B) The results showed that IFITM4P did not show any apparent effect on e the expression of TAK1 and SASH1.
(C) Phosphorylation of TAK1(Thr187) was obviously enhanced by IFITM4P dose (0-4 ng) in Leuk-1 cells, but phosphorylation of TAK1(Thr412) was not apparently changed.
(D) Knockdown of SASH1 in Leuk-1-IFITM4P repressed the mRNA of NF-κB p65. Data from A,B,D were shown as mean ± s.d. from three independent. *P < 0.05.

Supplementary Figure. 10

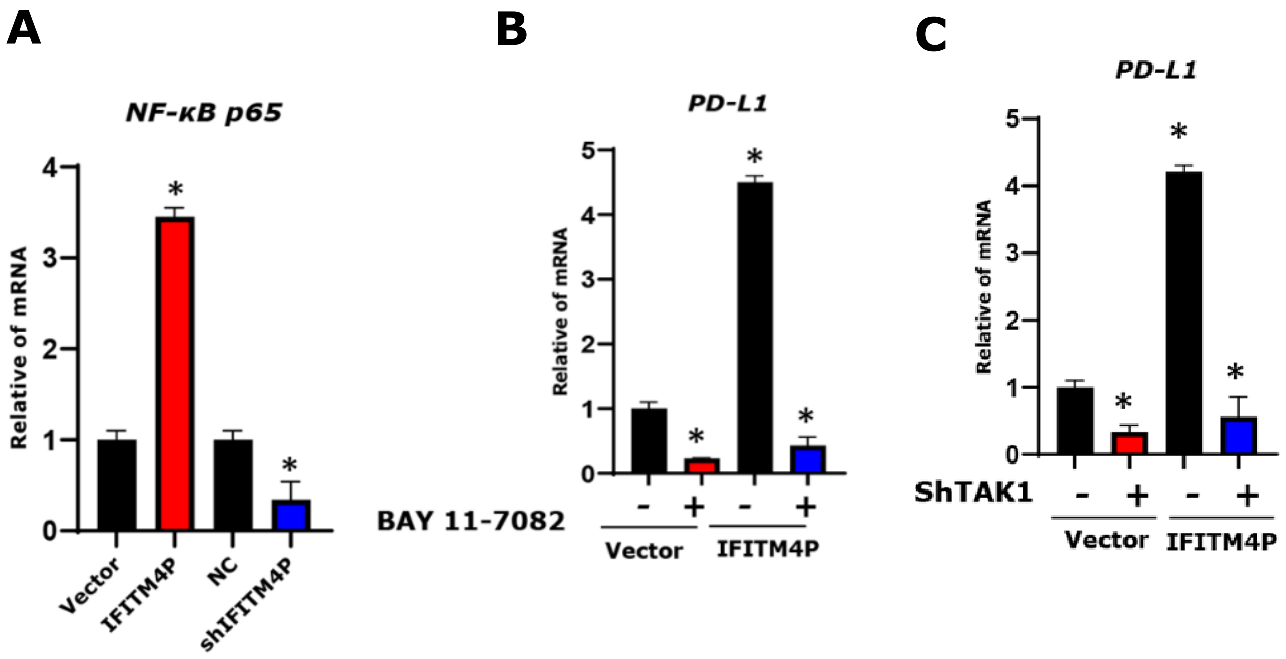

(A) NF-κB p65 levels are increased by the ectopic expression of IFITM4P in Leuk-1 cells and decreased by its knockdown.

(B) qRT-PCR shows repression of *PD-L1* transcription in BAY 11-7082 (10μM)-treated Leuk-1 cells, expressing either vector or IFITM4P.

(C) qRT-PCR analysis show that knockdown of TAK1 with shTAK1 repressed *PD-L1* transcription in IFITM4P expressing Leuk-1 cells.

Data from A,B,C were shown as mean ± s.d. from three independent.

*P < 0.05.

Supplementary Figure. 11

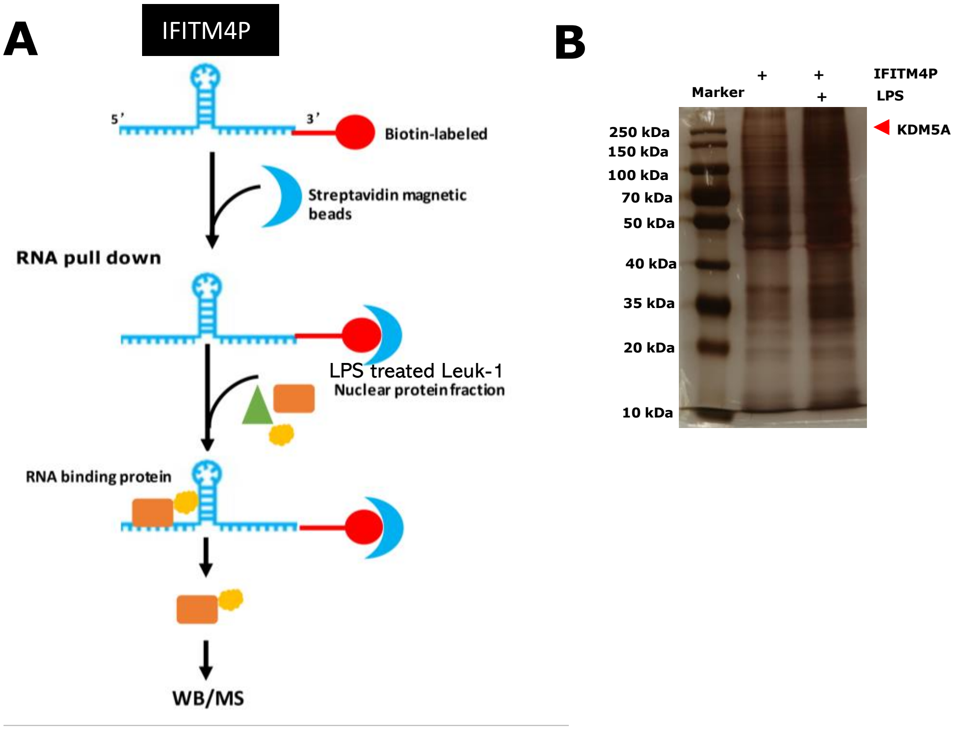

（A）Schematic of the RNA pulldown experiment for the identification of proteins associated with IFITM4P.

(B)　Leuk-1 with stable overexpression of IFITM4P was treated with LPS for 12 h. IFITM4P and its associated complexes were enriched by IP with streptavidin magnetic beads.

Supplementary Figure. 12

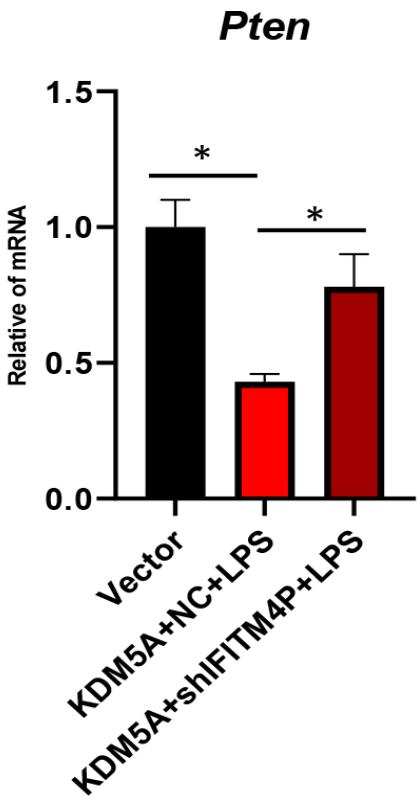

RT-PCR analysis of relative *Pten* mRNA expression in Vector and KDM5A-overexpressing Leuk-1 cells under LPS (100 μg/ml) treatment. Data were shown as mean ± s.d. from three independent. *P < 0.05.

Supplementary Figure. 13

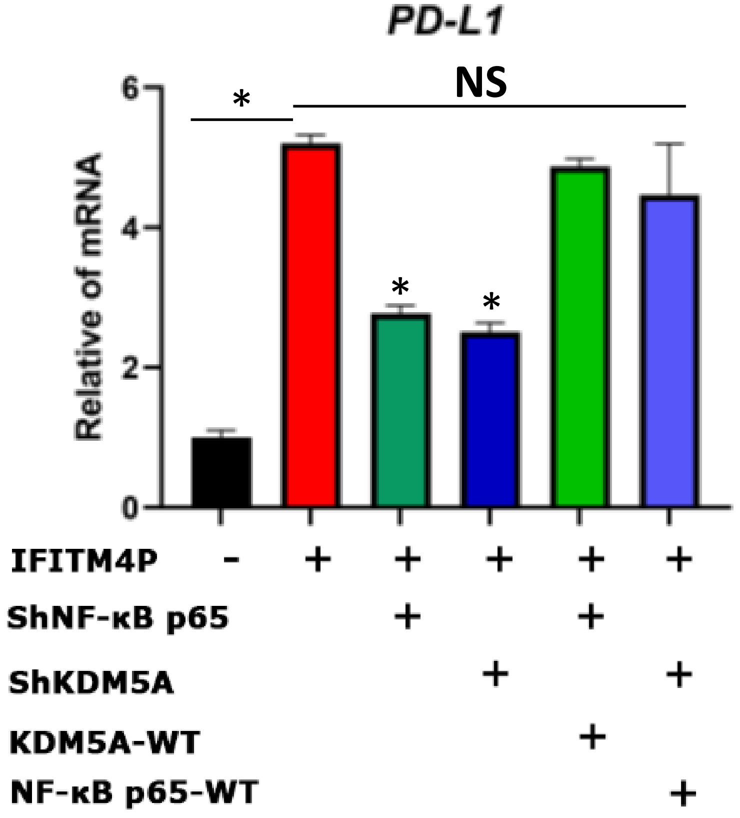

IFITM4P-HN4 and Vector-HN4 cells transiently transfected with ShRNAs to NF-κB p65 or KDM5A or scrambled control (ShRNA-NC), KDM5A or NF-κB p65, vector were treated with LPS (100 μg/ml). qRT-PCR showed a significant decrease in PD-L1 expression following the knockdown of KDM5A, and NF-κB p65, while KDM5A or NF-κB p65 increased the expression of PD-L1. Data were shown as mean ± s.d. from three independent. *P < 0.05. NS = no significant difference. WT = wild type.

Supplementary Figure. 14

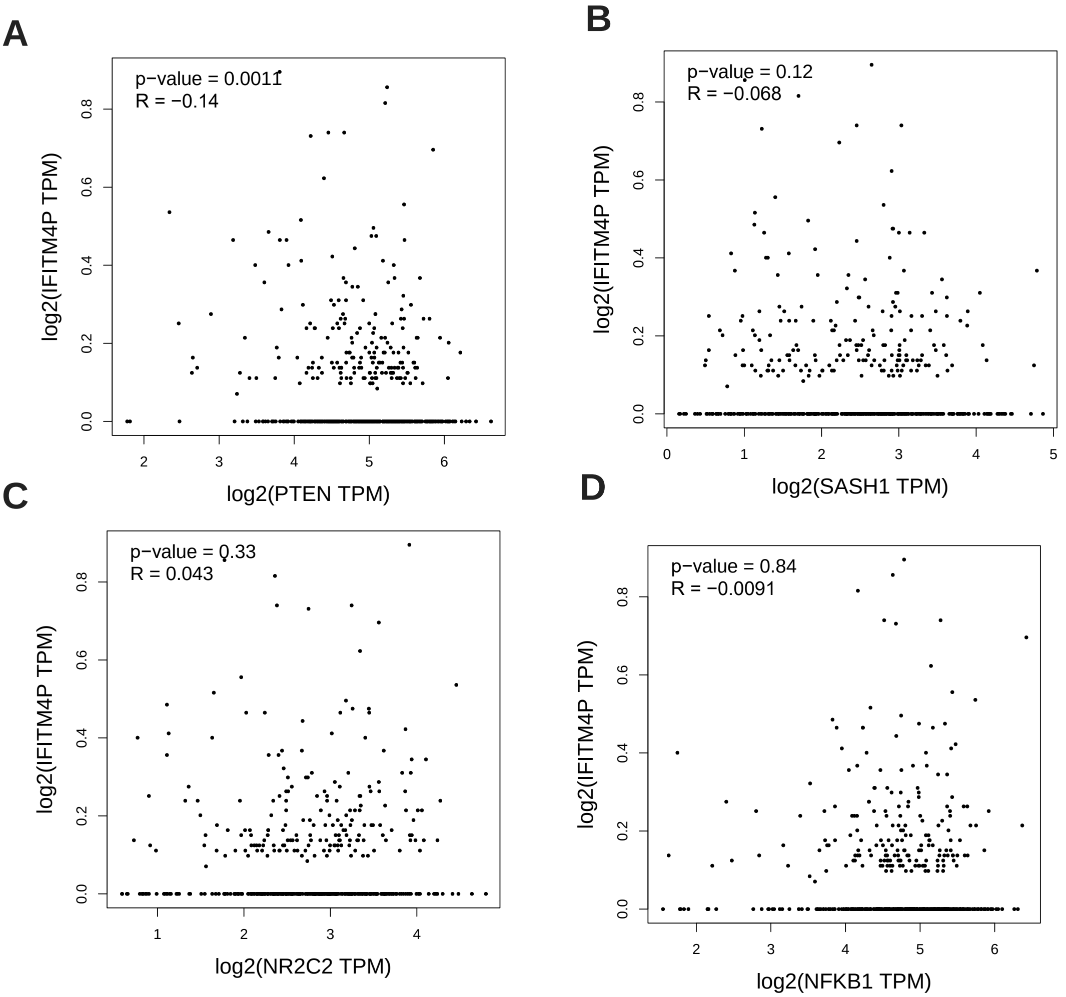

Data from The Cancer Genome Atlas (TCGA) indicated negative correlation between the levels of IFITM4P and PTEN (A) in HNSC samples (n = 518) (*P < 0.05), but no correlation between the levels of IFITM4P with SASH1(B), NR2C2(TAK1)(C), and NFKB1(D).

Supplementary Table 1. Clinicopathological information of patients in LncRNA microarray experiment

Number	sex*	age	site	Diagnosis**	epithelial dysplasia	OL-staging	OSCC-staging
1	F	76	tongue	NM	/	/	/
2	F	66	tongue	NM	/	/	/
3	F	46	gingiva	NM	/	/	/
4	M	41	tongue	OL	mild	III	/
5	F	51	tongue	OL	mild	III	/
6	F	47	tongue	OL	none	II	/
7	M	37	tongue	OL	mild	III	/
8	M	39	tongue	OSCC	/	/	II
9	M	48	tongue	OSCC	/	/	II
10	F	72	tongue	OSCC	/	/	II
11	M	55	tongue	OSCC	/	/	II
12	M	66	tongue	OSCC	/	/	II

* Sex: F, female; M, male

** NM, normal mucosa; OL, oral leukoplakia; OSCC, squamous cell carcinoma

Supplementary Table 2. Baseline characteristics of the patients enrolled in qRT-PCR validation of *IFITM4P* and *PD-L1* expression

	**NM group**	**OL group**	**OSCC group**	***P***
	N（%）	N（%）	N（%）	
sex				
female	13（56.52%）	34（50.75%）	15（32.61%）	0.084
male	10（43.48%）	33（49.25%）	31（67.39%）	
age				
mean ± sd	48.957±13.4342	58.194±13.4268	67±12.0831	＜0.001
smoking				
never	12（52.17%）	37（55.22%）	26（56.52%）	0.511
present or past	8（34.78%）	28（41.79%）	12（26.09%）	
N/A	3（13.04%）	2（2.99%）	8（17.39%）	
alcohol intake				
never	13（56.52%）	41（61.19%）	25（54.35%）	
present or past	0	24（35.82%）	13（28.26%）	1.000
N/A	10（43.48%）	2（2.99%）	8（17.39%）	
OL lesion area（mm^2）				
＜200		37（55.22%）		
≥200		27（40.30%）		
NA		3（4.48%）		
OL site				
Others	16（69.57%）	42（62.69%）		0.621
Lateral/Ventral Tongue	7（30.43%）	25（37.31%）		
lesion type				
homogeneous		45（67.16%）		
nonhomogeneous		22（32.84%）		
OL-staging				
I		10（14.93%）		
II		3（4.48%）		
III		45（67.16%）		
IV		9（13.43%）		
OSCC site				
non tongue			31（67.39%）	
tongue			15（32.61%）	
nerve invasion				
no			38（82.61%）	
yes			8（17.39%）	
histologic differentiation*				
well			37（80.43%）	
moderate			9（19.57%）	
poor			0（0%）	
OSCC-clinical staging				
I			9（19.57%）	

II	28（60.87%）
III	7（15.22%）
IV	2（4.35%）

Supplementary Table 3

Reagent or Resource	Source	Identifier
**Antibodies**		
PD-L1 Rabbit mAb	Immunoway	YT6033
TLR4 Rabbit mAb	Abcam	AB_2835322
KDM5A Rabbit mAb	Abcam	AB 70892
SASH1 Rabbit mAb	Immunoway	YT7427
TAK1 Rabbit mAb	Cell Signaling technology	Cat# 5602S
HA Rabbit mAb	Santa Cruz	Cat# sc-7392
TAK1 Rabbit mAb	Cell Signaling technology	Cat# 5602S
Phospho-TAK1 (Thr187) Antibody	Cell Signaling technology	Cat# 4536
Phospho-TAK1 (Thr412) Antibody	Cell Signaling technology	Cat# 9339
GAPDH Rabbit (D16H11)	Cell Signaling technology	Cat#5174
Phospho-NF-κB p65 (Ser536) mouse mAb	Cell Signaling technology	Cat# 3036
NF-κB p65 Rabbit mAb	Cell Signaling technology	Cat# 8242
Anti-Myc mAb	Cell Signaling technology	Cat #2276
**Chemicals**		
Lipofectamine 3000	Thermo SCIENTIFIC	Cat#L3000001
puromycin	Sigma Aldrich	Cat#P8833
BAY 11-7082	MCE	Cat#HY-13453
LPS	MCE	Cat#HY-D1056
TAK-242	MCE	Cat#HY-11109
Polymyxin B sulfate	MCE	Cat#HY-A0248
**Oligonucleotides** Primers; See Table S5		
**Critical Commercial Assays**		
Pierce TM Magnetic RNA-Protein Pull-Down Kit	Thermo SCIENTIFIC	Cat#20164
Malachite Green Phosphate Assay Kit	Cayman Chemical	Cat#1009325-96
Reverse Transcriptase Kit	Thermo SCIENTIFIC	Cat#18080044
T7 Transcription Kit	Thermo SCIENTIFIC	Cat#1354
RNAscope 2.0HD probe detection Kit	Advanced cell diagnostics	Cat#564781
SimpleChIP® Enzymatic Chromatin IP Kit (Agarose Beads)	Cell Signaling technology	Cat #9002
**Experimental Models: Cell Lines**		
Human: Leuk-1	American Type Culture Collection(ATCC)	
Human: HN4	American Type Culture Collection （ATCC）	
Human: 293T	American Type Culture Collection （ATCC）	
**Experimental Models: Organisms**		
B6/C57J	Shanghai SLAC Laboratory Animal Co.,Ltd	TD.09152

Supplementary Table 4

No.	Gene Symbol	GeneBank	Forward primer	Reverse primer	Ta(℃)
1	IFITM4P	NR_001590.1	CACTGCCCAAACCTTCTT	TGCTCCTCCTTGAGCATC	55
2	β-actin	DQ407611.1	CATTCCAAATATGAGATGCGTT	TACACGAAAGCAATGCTATCAC	60
3	TAK1	AF218074.1	GGATCCGGGATCATGTCGACAGCCTCCGC	CGCGGTACCTGAAGTGCCTTGTCGTTTCTG	55
4	SASH1	NM_015278.3	ATACCTCGGCTTGACATT	ATACCTCGGCTTGACATT	60
5	PD-L1	NM_001267706	TGCCGACTACAAGCGAATTACTG	CTGCTTGTCCAGATGACTTCGG	55
6	NF-KB P65	NM_021975.3	ATGCGCTTCCGCTACAAGTG	ACAATGGCCACTTGTCGGTG	55
For ChIP	P1		GCTTAGGTCTCAACTCAGA	CATCAACTCCAATGTAGGTAG	58
	P2		GGCTGTACGCTAGTTATCA	GTTAGGCTTACCGATGTTG	58
